# Host selection is not a universal driver of phyllosphere community assembly among ecologically similar native New Zealand plant species

**DOI:** 10.1186/s40168-024-02000-x

**Published:** 2025-01-31

**Authors:** Anya S. Noble, Jaber Abbaszadeh, Charles K. Lee

**Affiliations:** School of Science, University of Waikato, Hamilton, New Zealand

**Keywords:** Phyllosphere microbiome, Community assembly, Host selection, Microbial dispersal, *Leptospermum scoparium*, Epiphytic bacterial communities, Leaf surface

## Abstract

**Background:**

A growing body of evidence demonstrates that host-associated microbial communities of plant leaf surfaces (i.e. the phyllosphere) can influence host functional traits. However, it remains unclear whether host selection is a universal driver of phyllosphere community assembly. We targeted mānuka (*Leptospermum scoparium*) and three neighbouring non-mānuka plant species along an 1800-m transect in a New Zealand native bush to conduct a hypothesis-driven investigation of the relative influence of host species identity and stochastic dispersal on the composition of natural phyllosphere bacterial communities.

**Results:**

We detected significant correlations between host species identity and mānuka phyllosphere communities that are consistent with a dominant role of host selection in the assembly of the mānuka phyllosphere microbiome. In contrast, the phyllosphere community compositions of neighbouring, ecologically similar native plants were highly variable, suggesting that stochastic processes, such as dispersal, had a stronger influence on the phyllosphere microbiomes of those non-mānuka plants compared to the phyllosphere microbiome of mānuka. Furthermore, the distribution of phyllosphere taxa among plant species was congruent with a scenario in which microorganisms had dispersed from mānuka to non-mānuka phyllosphere microbiomes.

**Conclusions:**

We conclude that host selection of phyllosphere communities is not and should not be presumed to be a universal trait across plant species. The specificity of the mānuka phyllosphere microbiome suggests the presence of functionally significant bacteria that are under direct, possibly chemically mediated, selection by the host. Furthermore, we propose that phyllosphere microbiomes under strong host selection, such as that of mānuka, may act as a source of microorganisms for the phyllosphere microbiomes of neighbouring plants.

Video Abstract

**Supplementary Information:**

The online version contains supplementary material available at 10.1186/s40168-024-02000-x.

## Background

Plant leaf surfaces are colonised by diverse communities of epiphytic microorganisms, defined as the phyllosphere microbiome [1, 2]. Despite having received limited attention from microbial ecologists until recent years, a growing body of evidence from laboratory model plant species has demonstrated that reciprocal interactions with epiphytic bacterial communities can have a significant influence on host plant functional traits [3–6]. Such microbiome-mediated host functional traits include increased disease resistance [3, 5], nutrient acquisition [7, 8], and stress tolerance [6]. Gaining a fundamental and generalisable understanding of the processes that shape the assembly of epiphytic bacterial communities is required to identify and promote beneficial compositions, which is especially desirable for economically and environmentally significant plant species.

Although there is a steadily growing body of empirical data on the ‘phyllosphere microbiome’, our understanding of epiphytic community assembly is severely limited by the broad and inconsistent use of the term ‘phyllosphere’. In the broadest sense, ‘phyllosphere’ has been used to refer to the epiphytic and endophytic microbial communities associated with all aerial plant structures, including leaves, stems, bark, flowers, and fruit [9, 10]. In many other cases, ‘phyllosphere’ has been used to describe only the epiphytic communities associated with all aerial plant structures [11] or the epiphytic and endophytic microorganisms extracted from only leaves [12–14]. Given that different plant structures represent ecologically distinct habitats and harbour ecologically distinct microbiomes [15–18], coarse sampling approaches (i.e. pooling functionally explicit communities into a single sample) encouraged by these less stringent definitions of ‘phyllosphere’ are likely to obscure the detection of microbiome-specific ecological processes [19]. In addition, inexact terminology promotes methodological inconsistencies that further hinders generalisability across studies. For example, studies that include endophytic microorganisms often use chloroplast-excluding 16S rRNA gene PCR primers due to the presence of large quantities of host plastids released during the maceration of plant tissue [12]. In comparison, studies that exclusively target epiphytes are more likely to use universal primers as plant tissue is not macerated during sample processing [20]. As a result, the body of ‘phyllosphere microbiome’ literature is growing haphazardly on a foundation of conceptual and empirical ambiguity. Defining and sampling habitats at microbiologically relevant scales remains essential in order to study specific ecological processes and host-microbiome associations. Herein, we define the phyllosphere *sensu stricto* as the epiphytic microbial communities present on the leaf surface and only refer to studies that adhered with this original definition.

The phyllosphere microbiome is continuously exposed to bacteria that disperse in the near-surface atmosphere from distant and local sources, such as soil and vegetation [21]. Nevertheless, the communities that establish on the leaf surface are generally distinct from those in the surrounding environment [22, 23]. In particular, host species- and even genotype-specific patterns of community composition have been widely observed, from which a dominant role of host selection in phyllosphere community assembly has been inferred [24–27]. However, the relative strength of host selection in structuring phyllosphere community composition remains enigmatic. For example, some studies have found that geographic region has a larger influence on the phyllosphere microbiome than host species identity, implying that phyllosphere communities can also be predominantly structured by stochastic processes, such as dispersal limitations, or regional environmental conditions [28, 29]. Although methodological differences (including spatial scale) may account for a significant proportion of this inconsistency, it is also probable that the drivers of phyllosphere microbiome assembly are not universal across plant species [30]. Some studies have presumed significant host selection across all plant species in their experimental design or data interpretation, which potentially undermined the robustness and generalisability of their findings [31, 32]. Evaluating the universality of host selection in phyllosphere microbiome assembly requires careful control of environmental variables, yet it is also critically important that such studies focus exclusively on stable, ideally natural, specimens (e.g. perennial evergreens) since priority effects may have an overwhelming influence on simplified laboratory experimental systems that do not reach a steady state [33, 34].

The decline in community similarity with increasing geographic distance, or distance-decay relationship, is a widely recognised ecological pattern that represents a useful tool to test ecological theories, such as the relative role of selection versus dispersal in microbial community assembly [35–37]. For example, in a scenario in which community assembly is strongly influenced by dispersal limitation (relative to local selection), ecologically equivalent communities may become increasingly dissimilar in their composition as the physical distance between them increases [38]. Meanwhile, ecologically discrete communities may become more similar in their composition as the physical distance between them decreases [39]. In contrast, a relatively weaker distance-decay relationship is more likely to be observed in a scenario in which community assembly is strongly influenced by local selection (relative to dispersal) [38]. Although a role of dispersal limitation in phyllosphere community assembly has been indirectly inferred from observations of large-scale biogeographic patterns in community composition [28], only two studies have systematically investigated the effect of spatial distance in the phyllosphere (i.e. by employing sampling strategies to minimise confounding environmental heterogeneity). One study sampled the *Tamarix aphylla* phyllosphere microbiome along a 500-km transect in the Sonoran Desert and reported a significant relationship between distance and Betaproteobacteria [29]. Another study sampled the *Magnolia grandiflora* phyllosphere microbiome that was separated by distances of up to 450 m in a 20-ha Mississippi forest plot and identified a significant relationship between distance and total phyllosphere community dissimilarity [40]. More generally, the role of stochastic processes in phyllosphere community assembly has also been examined in controlled experiments using annual model plants or crops [34, 41]. Although the effect of confounding environmental variables is generally reduced in an experimental setting, these results are not necessarily generalisable to natural communities nor perennial plant species [34]. Sampling natural phyllosphere communities at exponentially increasing distances will be necessary to test whether the stochastic dispersal of phyllosphere bacteria conforms to the exponential model of distance-decay [36, 37]. Furthermore, deliberately targeting conspecific and heterospecific phyllosphere samples will be important to shed light on the relative contribution of dispersal versus host selection among different plant species.

*Leptospermum scoparium* J. R. Forst et G. Forst, commonly known as ‘mānuka’, is a culturally and economically significant flowering tea tree species, indigenous to Aotearoa New Zealand [42]. Honey derived from the nectar of the mānuka tree contains unique non-peroxide antibacterial properties and has become a highly valuable commodity [42, 43]. The unique antibacterial properties originate in the nectar of the mānuka flower due to the accumulation of a three-carbon sugar called dihydroxyacetone (DHA), which undergoes a chemical conversion to the main antibacterial constituent, called methylglyoxal (MGO), in mature honey [44, 45]. However, the quantity of DHA that accumulates in the nectar of the mānuka flower is notoriously variable, consequently causing large regional and annual fluctuations in the antimicrobial efficacy of mānuka honeys [46, 47]. Despite extensive research efforts, no reliable correlation has been identified between DHA production and climate [47], soil properties [48], host genetics [49], fungi [50], or endophytes [51, 52]. In a previous study, we characterised the phyllosphere bacterial community structure of geographically distant and environmentally diverse populations of mānuka and identified a dominant and ubiquitous core phyllosphere microbiome [53]. Such specific host association patterns have seldom been observed in the phyllosphere of other host species and are congruent with those of a microbial community under strong host selection [54, 55]. However, the host specificity of the mānuka phyllosphere microbiome remains to be determined, particularly in relation to phyllosphere microbiomes of physiologically and/or ecologically similar plants native to New Zealand.

In the present study, we used a multi-species, spatially hierarchical sampling design to systematically investigate whether the mānuka phyllosphere microbiome is primarily influenced by host species identity or dispersal in the absence of confounding environmental gradients. Specifically, we sampled focal mānuka and a neighbourhood of three non-mānuka plant species at replicate sites separated by quasi-exponentially increasing distances (i.e. 4, 16, 80, 400, and 1800 m) along an 1800-m transect of native vegetation. Given the close host-microbiome association previously identified in the mānuka phyllosphere, we hypothesised that the assembly of the mānuka phyllosphere microbiome is overwhelmingly driven by host selection relative to the effects of local stochastic processes. To evaluate this, we addressed the following questions:

1. Do phyllosphere bacterial taxa disperse stochastically among mānuka and neighbouring phyllosphere microbiomes (i.e. does the distribution of phyllosphere taxa within each site reflect a random probability distribution)?
2. Is the mānuka phyllosphere microbiome colonised by specific bacterial communities that are distinct from ecologically similar plant neighbours?
3. Do patterns of host species identity or distance-decay prevail in the phyllosphere bacterial communities of these four plant species?
4. Does the contribution of surface soil bacteria to the phyllosphere microbiome differ among plant species?

## Methods

### Study site

This study was carried out on lands administered by the Lake Rotoaira Forest Trust adjacent to the Dual World Heritage Tongariro National Park in the Central North Island of New Zealand at an altitude of 800 m ASL (39°10 S; 175°46 E). This site was located on a subalpine plateau and comprised a total land area of 660.9 ha of native vegetation, of which 31.7% is forest and 68.1% is dense shrubland dominated by mānuka (*Leptospermum scoparium*) and kānuka (*Kunzea ericoides*) [56–59]. The mean annual temperature at the nearest weather station at Turangi (39.0 S, 175.8 E, 366 m ASL, 17 km from site) is approximately 12 °C and mean annual rainfall is approximately 1600 mm. Within this site, a single 1800-m northeast transect was established across an area of undisturbed mānuka/kānuka dominated scrub that exhibited visually homogenous topography and vegetation. Specifically, elevation was constant (i.e. 800 m ASL) across the length of this transect and no climatic gradient was apparent. Furthermore, no changes in forest age, density, diversity, or canopy height were observed along the transect.

### Plant species characteristics

In addition to mānuka, three native non-mānuka plant species were selected for study. These included kānuka (*Kunzea ericoides*), tawiniwini (*Gaultheria antipoda*), and toatoa (*Phyllocladus alpinus*). These neighbouring plant species were chosen because of their ecological and morphological similarity to mānuka. Specifically, all plant species were woody perennial evergreens that possess xeromorphic adaptations and naturally grow within the same shrub stratification layer. These three plant species were also chosen such that the non-mānuka leaf samples ranged in morphological similarity to mānuka. Mānuka (family Myrtaceae) has small (4–12 mm long) and narrow leaves that have a low leaf area index and a sharp point (Supplementary Fig. 1A). In addition, mānuka leaves are commonly described as xeromorphic, featuring adaptations such as a thick waxy cuticle (> 10 µm) (see Supplementary Table 1 for more details) [60]. Mānuka trees that were selected for sampling ranged in height from 2.6 to 3.6 m. Kānuka (family Myrtaceae) is a bushy, evergreen shrub or tree that has leaves that are small (4–12 mm long) and narrow with a low leaf area index, similar to mānuka leaves, but with a reduced point (Supplementary Fig. 1B). Kānuka exhibits many ecological and morphological similarities to mānuka and was formally known as *Leptospermum ericoides* until 1983 [61]. However, despite these superficial similarities, kānuka is in fact phylogenetically distinct from mānuka (see Supplementary Table 1 for more details) [62]. Kānuka trees selected for sampling ranged in height from 2.6 to 6.4 m. Tawiniwini (family Ericaceae) has rounded (5–15 mm long), leathery, and toothed leaves (Supplementary Fig. 1C, see Supplementary Table 1 for more details). The tawiniwini plants selected for sampling ranged in height from 0.9 to 1.8 m. Toatoa (family Podocarpaceae) is a strongly aromatic gymnosperm shrub that is distinguished by the presence of phylloclades, flattened stem structures (5–25 mm long) that simulate the form and function of a foliage leaf (Supplementary Fig. 1D, see Supplementary Table 1 for more details). Phylloclades are considered xeromorphic and are the only specialised photosynthetic organs in mature *Phyllocladus* plants [63, 64]. Given their morphological and functional equivalence to leaves, the phylloclades on toatoa were sampled and processed synonymously with the leaf samples of the other plant species. Toatoa trees that were selected for sampling ranged in height from 2 to 6 m. See Supplementary Table 1 for further plant species descriptions.

### Sample collection

**Fig. 1:**
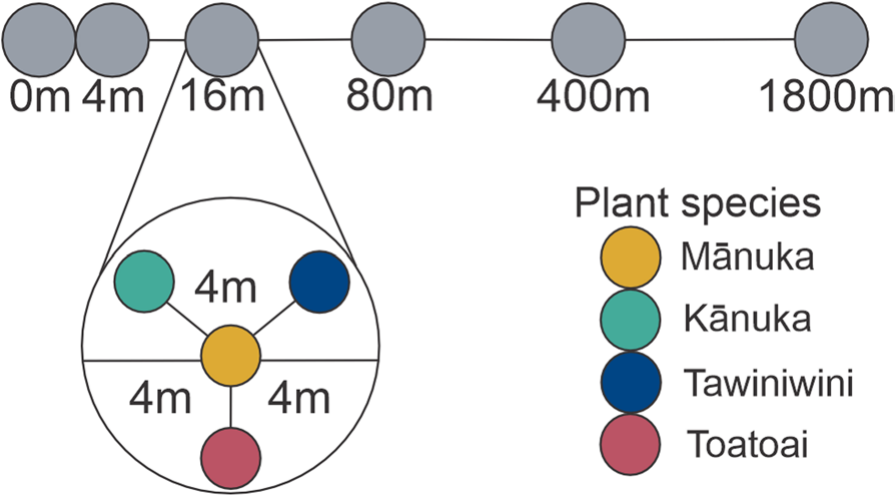
Schematic representation of transect sampling design. From the point of origin (0 m), each consecutive transect sample site (grey) was separated by quasi-exponentially increasing distances. Within each replicate site, mānuka and three non-mānuka plant species were sampled (inset). Non-mānuka plant species were approximately 4 m from the focal mānuka. Colour represents host species identity

Within a single day in the summer of 2021, branch and surface soil samples were collected from a focal mānuka and three surrounding non-mānuka plant neighbours. Neighbouring plant species were kānuka (*Kunzea ericoides*), tawiniwini (*Gaultheria antipoda*), and toatoa (*Phyllocladus alpinus*), and one of each of these species was sampled approximately 4 m from the focal mānuka (Fig. 1). This sampling design was replicated at six sites along the 1800-m transect, separated by quasi-exponentially increasing distances ranging from 4 to 1400 m (Fig. 1). These distances (to the point of origin) were chosen because distance-decay curves are best fit by negative exponential functions. Three branches per focal mānuka and one branch per neighbouring non-mānuka plant species were cut with clippers sterilised on site with 70% *v/v* ethanol/water, placed in individual sterile zip lock bags, and immediately placed on dry ice. Sampled branches were mature (i.e. established woody), seemingly healthy with no obvious signs of herbivory or disease, and similarly sized. Furthermore, since the spatial position of branches within individual trees may drive variation in phyllosphere community composition—either due to differences in the accessibility of leaves to dispersing microorganisms or small-scale environmental conditions—branches were sampled at a relatively constrained range of heights (mānuka: 2–3.8 m, kānuka: 1.6–3.6 m, tawiniwini: 0.7–1.6 m, toatoa: 1–2.35 m) and aspects (i.e. NW). Surface soil (1–2 mm) from around the base of each tree was collected into sterile 50-mL Falcon tubes using a spatula sterilised on site using 70% *v/v* ethanol/water and immediately placed on dry ice. Upon return to the Thermophile Research Unit at the University of Waikato, branch and soil samples were frozen at − 20 °C until further analysis. Several attributes of each sample tree were measured during sampling. These included tree height, tree basal diameter, and the distance of the focal mānuka to each of the three non-mānuka plant neighbours, as well as the height and aspect of each branch collected. GPS coordinates (WGS84 (G1762) in decimal minutes) were determined for each tree at the time of sampling.

### DNA extraction, amplification, and sequencing

Per branch, 1 g of healthy, undamaged green leaves was carefully and aseptically excised and pooled. Leaves that exhibited distinct characteristics of juvenile foliage were excluded. Epiphytes were recovered from the surface of excised leaves using a sonication protocol that has been previously described [53]. Briefly, leaves were sonicated in 10 mL of phosphate buffer wash solution (100 mM NaH2PO4, 1% tween 20) for 10 min using an ultrasonic cleaning bath (60 Hz). After sonication, the wash solution was decanted and the sonicated leaves were submerged and rinsed with another 10 mL of PBS. This rinse step was repeated twice. The total wash solution (30 mL) was syringe filtered (90 µm) to remove fine plant debris and centrifuged (3200 × g) for 30 min. The pellet was resuspended in 270 µL of PBS, transferred to a 2.0-mL bead tube containing 0.5 g each of 0.1 mm and 2.5 mm silica-zirconia beads, and frozen at − 80 °C until DNA extraction. Total epiphytic DNA was extracted from sonicated resuspensions using a modified cetyl trimethylammonium bromide (CTAB) bead-beating protocol, which has been proven to be highly effective for low biomass samples [65]. For each surface soil sample, total DNA was extracted from 0.5 g of soil using the Power Soil DNA Extraction kit (QIAGEN). DNA was quantified using the Qubit dsDNA HS Assay Kit (Thermo Fisher Scientific, USA) and stored at − 20 °C until further analysis. The V4-V5 hypervariable region of the 16S rRNA gene was amplified with 515FY/926R fusion primers (5′GTGYCAGCMGCCGCGGTAA/5′CCGYCAATTYMTTTRAGTTT) and previously described PCR conditions in a one-step protocol to minimise cross-contamination of aerosolised PCR products [53]. PCR products were cleaned and normalised with SequelPrep™ (Thermo Fisher Scientific). Normalised samples were pooled at an equimolar concentration into two libraries. An Illumina MiSeq 300PE sequencing run was performed on each library at Massey Genome Service (Palmerston North, New Zealand).

### Sequence quality control and taxonomic assignment

The raw 16S rRNA gene amplicon sequences were processed using the DADA2 pipeline [66]. Forward and reverse reads were truncated at 237 and 232 bp, respectively, and quality filtered using the ‘filterAndTrim’ function with the following settings: maxN = 0, maxEE = c(3, 3), and truncQ = 2. Error rates were determined with the ‘learnErrors’ function and used to remove sequencing errors from forward and reverse reads, which were then assigned to amplicon sequence variants (ASVs) using the ‘dada’ function. Paired reads were then merged, converted into an ASV table, and chimaeras removed with the removeBimeraDenovo function using the method ‘consensus’. Taxonomy was assigned using the ‘assignTaxonomy’ and ‘addSpecies’ functions using the native implementation of the naive Bayesian classifier and the SILVA database version 138.1 [67]. Chloroplast and mitochondrial sequences were filtered out by removing all ASVs with a taxonomic assignment of ‘Chloroplast’ at the order level and ‘Mitochondria’ at the family level, respectively. Lastly, we applied the ‘isContaminant’ function (method = prevalence) from the package ‘decontam’ to our samples using our blank DNA extractions and PCR reactions to identify and remove putative contaminants introduced during processing [68].

After 16S rRNA gene sequence reads were quality filtered, one sample (Rp_03.1) stood out as an outlier. This sample was first identified due to its uniquely large number of sequencing reads (43,184) relative to all other samples (< 27,000) (Supplementary Fig. 2A). This sample was dominated by the genus *Sodalis*, which comprised 32 ASVs and represented 62.4% of the reads of this sample (Supplementary Fig. 3). In comparison, *Sodalis* comprised only 0.25% and 0.57% of the reads in two other samples originating from the same tree and was entirely absent in all other samples. The genus *Sodalis* contains several insect endosymbionts and is thus very likely not a part of the native microbial phyllosphere population [69]. Therefore, sample Rp_03.1 was removed from further analyses and 17,315 ± 4128 quality-assured reads per sample remained.

### Data analysis

All statistical analyses were conducted in R (R Core Team, 2022). Alpha diversity analyses were conducted using the ‘vegan’ package [70]. Each sample was subsampled 100 times to an even sequencing depth (6192 reads) and the average richness and Shannon–Wiener index was calculated for each sample. Pairwise Bray–Curtis and Jaccard community dissimilarities were calculated using the ‘vegdist’ function on ASV relative abundance and presence/absence transformed data, respectively. The following analyses were carried out to address each of our four research questions.

- Q1: Do phyllosphere bacterial taxa disperse stochastically among mānuka and neighbouring phyllosphere microbiomes (i.e. does the distribution of phyllosphere taxa within each site reflect a random probability distribution)?

**Fig. 2:**
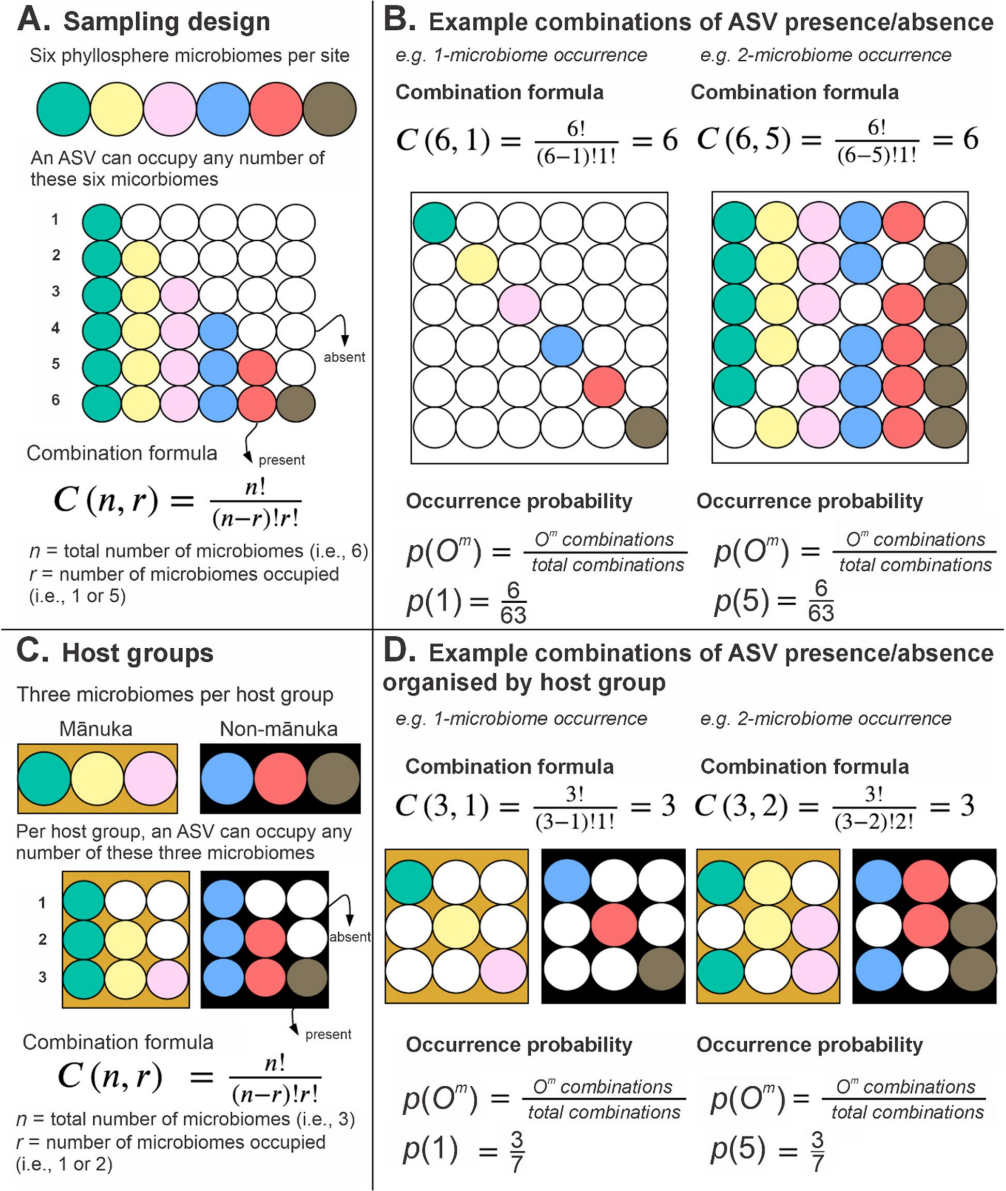
Combinatorial schematic and occurrence probability approach. **A** Each ASV has the potential to colonise any number of the six spatially structured phyllosphere microbiomes (i.e. three mānuka and three non-mānuka samples) per site (termed microbiome occurrence, O^m^). For each microbiome occurrence, the number of unique combinations of ASV presence/absence is calculated using the combination formula. The total number of possible combinations for all microbiome occurrences (1–6) is 63. The probability that an ASV is present at a microbiome occurrence of *N* is calculated by dividing the number of unique combinations for *N* microbiome occurrence by the total number of combinations. **B** For example, ASVs that are present in either one or five microbiomes can each exhibit six unique combinations of presence/absence. Therefore, the corresponding occurrence probability for each of these microbiome occurrences is 6/63. **C** The abstraction of two host groups, ‘mānuka’ and ‘non-mānuka’, was used to further organise the six phyllosphere microbiomes within each site. In this scenario, each ASV colonises any number of the three phyllosphere microbiomes per host group. For each microbiome occurrence, the number of unique combinations of ASV presence/absence is calculated using the combination formula. The total number of possible combinations for all microbiome occurrences per host group (1–3) is seven. The probability that an ASV is present in a host group at a microbiome occurrence of *N* is calculated by dividing the number of combinations of *N* microbiome occurrence by the total number of combinations. **D** For example, ASVs that are present in either one or two microbiomes per host group can each exhibit three unique combinations of presence/absence. Therefore, the corresponding occurrence probability for each of these microbiome occurrences is 3/7

Occurrence probabilities provide a quantitative description of the likelihood of an organism being present at a location as well as a mechanism for testing hypotheses related to factors that influence occurrence [71–73]. We used occurrence probabilities of taxa across the spatially structured phyllosphere microbiomes in our replicate transect sites to test hypotheses related to local dispersal. Assuming complete neutrality in the assembly of the phyllosphere microbiome (and assuming each tree at each site is exposed to a single homogenous pool of microorganisms), each ASV has the potential to exhibit one of 63 unique combinations of occurrence per site (corresponding to the unique combinations of presence/absence across each of the six microbiomes). The number of unique combinations of presence/absence were used to calculate zero-truncated probabilities that describe the likelihood of an ASV being present in any number of phyllosphere microbiomes (i.e. 1–6, herein termed *microbiome occurrence*) (see Fig. 2A–B for schematic explanation of how occurrence probabilities were generated, see Supplementary Table 2 for occurrence probabilities). Chi-square goodness-of-fit tests were used to determine whether the empirical presence/absence data (observed proportion of ASVs at each microbiome occurrence) differs from the distribution of occurrence probabilities, aka the predicted distribution. We next categorised the phyllosphere microbiomes as either ‘mānuka’ or ‘non-mānuka’. Organising the microbiomes as such provided two host groups of an equal number of spatially structured microbiomes per site (i.e. mānuka = 3, non-mānuka = 3). Using the same assumptions of neutrality, we then used the same approach as above to calculate zero-truncated probabilities that describe the likelihood of an ASV being present at each microbiome occurrence (i.e. 1–3) within each host group (see Fig. 2C–D for schematic explanation of how occurrence probabilities were generated for individual host groups, see Supplementary Table 5 for occurrence probabilities). Chi-square goodness-of-fit tests were used to determine whether the empirical presence/absence data differs from the distribution of occurrence probabilities. Jensen-Shannon divergence was used to calculate the distance between the empirical and predicted distributions for each host group. Lastly, we determined ‘preferential host occupancy’ for each ASV, defined as the host group in which any given ASV had the largest occupancy. For example, if an ASV was present in all three mānuka microbiomes and one non-mānuka microbiomes, mānuka was considered the preferential host. The number of ASVs with ‘mānuka’, ‘non-mānuka’, or ‘no’ preferential host occupancy was determined for each microbiome occurrence and compared to the ratios generated for the predicted distribution.

- Q2: Is the mānuka phyllosphere microbiome colonised by specific bacterial communities that are distinct from ecologically similar plant neighbours?

Species-specific core microbiomes were determined using a 100% prevalence method with the underlying assumption that the spatial persistence of core ASVs across ecologically equivalent communities implies an important role of these ASVs in host plant fitness [54, 55]. Indicator taxa analysis was also performed using the ‘multipatt’ function in the ‘indicspecies’ package [74]. Benjamini–Hochberg corrected Kruskal–Wallis tests were used to evaluate significant differences in taxon relative abundance (i.e. core/indicator) and host species identity.

- Q3: Do patterns of host species identity or distance-decay prevail in the phyllosphere bacterial communities of these four plant species?

The Kruskal–Wallis test was used to evaluate significant differences in alpha diversity (Shannon and richness) across host species. Differences in community structure among host species were then assessed using both an ANOSIM and PERMANOVA on community dissimilarities using the ‘adonis’ and ‘anosim’ functions in the ‘vegan’ package, respectively. Mantel tests were used to test the correlation between community dissimilarity and spatial distance. The normalised stochasticity ratio (NST) was used in the ‘NST’ package with the ‘EF’ null model to estimate ecological stochasticity of community assembly; 50% was taken as the boundary point between more deterministic (< 50%) and more stochastic (> 50%) assemblies [75].

- Q4: Does the contribution of surface soil bacteria to the phyllosphere microbiome differ among plant species?

The Kruskal–Wallis test was used to evaluate significant differences in alpha diversity (Shannon and richness) across sample types (i.e. phyllosphere and soil). The influence of host species identity and spatial distance on surface soil community dissimilarity was assessed using analyses previously described for Q3. The number of ASVs shared among phyllosphere and surface soil samples was assessed overall and individually for each host species. The presence of a surface soil core microbiome was investigated using a 100% prevalence threshold as previously described for Q2.

## Results

Phyllosphere samples were collected from focal mānuka (*L. scoparium*) and three neighbouring plant species, kānuka, tawiniwini, and toatoa, at six replicate sites separated by quasi-exponentially increasing distances along an 1800-m transect of native bush (Fig. 1). Neighbouring plant species were endemic to New Zealand and ranged in ecological and morphological similarity to mānuka (Supplementary Fig. 1, see Supplementary Table 1 for comparisons). Sequencing of bacterial 16S rRNA gene PCR amplicons from the phyllosphere of these plant species yielded 17,315 ± 4128 quality-assured reads per sample. In total, 4765 ASVs were identified at an average of 619 ± 120 ASVs per phyllosphere sample.

### Patterns of local dispersal differ between mānuka and non-mānuka neighbouring plant species

Experimental phyllosphere studies have suggested that phyllosphere community composition can be directly influenced by the dispersal of bacteria among plant neighbours, indicating phyllosphere microbiomes have the potential to act as both ‘source’ and ‘sink’ communities [41]. We therefore used occurrence probabilities (see ‘Methods’) and the observed distribution of taxon presence/absence to test a series of hypotheses aimed at tentatively inferring local patterns of dispersal within our six replicate transect sites.

H1: bacteria do not disperse between phyllosphere microbiomes (i.e. all six communities are sinks).

**Fig. 3:**
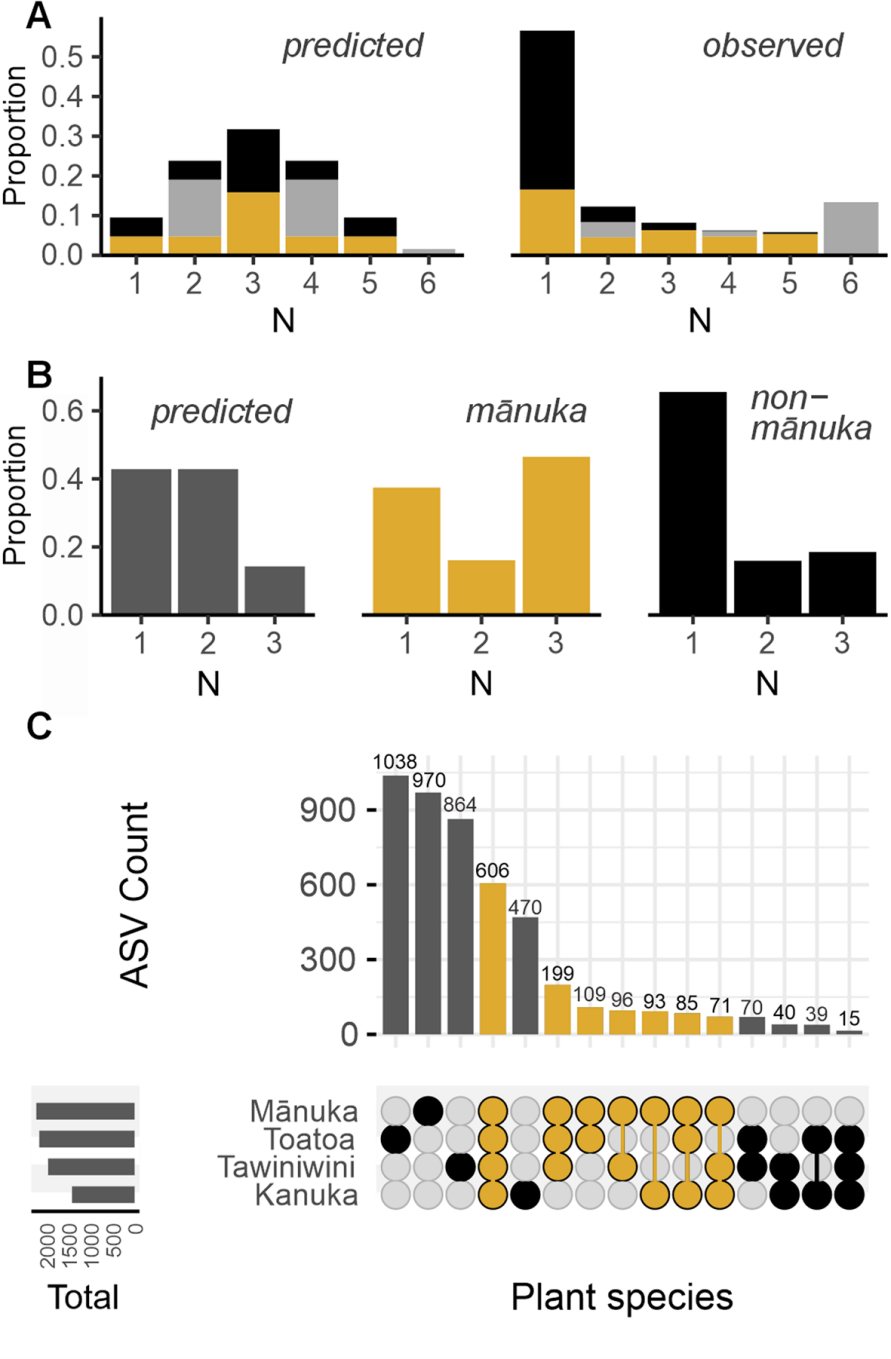
Predicted versus observed distribution of ASVs across the spatially structured phyllosphere microbiomes within each site. **A** Bar plots showing the proportion of ASVs predicted (left) versus the proportion of ASVs that were observed (right) for each microbiome occurrence (*N*). Within these bar plots, colour indicates the predicted (left) versus observed (right) proportion of preferential host occupancy: mānuka (yellow), non-mānuka (black), and no preference (grey). **B** Bar plots showing the proportion of ASVs predicted (left) versus the proportion of ASVs that were observed for each microbiome occurrence (*N*) within mānuka (middle) and non-mānuka (right) microbiome host groups. All proportions are averaged per site. **C** Upset plot demonstrating the total number of shared and unique ASVs across each possible host species microbiome combination. Yellow represents host species combinations that include mānuka

In a scenario of no inter-host dispersal, we would expect an entirely stochastic dispersal of bacteria from the surrounding environment to each of our six spatially structured phyllosphere microbiomes (i.e. ASV presence/absence to be well described by a random probability distribution). We tested for this scenario by comparing the empirical distribution of ASVs (i.e. the proportion of ASVs that were observed at each microbiome occurrence) with their corresponding occurrence probabilities (Supplementary Tables 2–3). We found that the empirical and predicted distribution of ASVs were significantly different (chi-square goodness of fit *p* < 0.001, Fig. 3A, see Supplementary Table 4 for *X*^2^ values). Compared to the predicted stochastic distribution, a larger proportion of ASVs were observed in a single microbiome. We also observed a larger proportion of ASVs present in all six microbiomes than what was predicted for a completely stochastic dispersal. These observations suggest that chance alone is not sufficient to describe the distribution of taxa, indicating the possibility of dispersal from neighbouring plants.

H2: bacteria do not disperse between mānuka or non-mānuka phyllosphere microbiomes (i.e. mānuka and non-mānuka communities are sinks).

We next looked for differences between groups of host species by testing for a stochastic distribution of taxa within ‘mānuka’ and ‘non-mānuka’ phyllosphere microbiomes. As for H1, we tested this by comparing the empirical distribution of ASVs with their corresponding occurrence probabilities (Supplementary Tables 5–6). We found that the observed distribution of ASVs in each of the mānuka and non-mānuka microbiome groups was significantly different to the predicted stochastic distribution (chi-square goodness of fit *p* < 0.001, Fig. 3B, see Supplementary Table 7 for *X*^2^ values). Compared to the predicted stochastic distribution, the non-mānuka phyllosphere had a larger proportion of ASVs that were present in only a single microbiome and a reduced proportion of ASVs that were present in two microbiomes (Fig. 3B). Meanwhile, the mānuka phyllosphere had a larger proportion of ASVs that were present in three microbiomes and a reduced proportion of ASVs that were present in two microbiomes (Fig. 3B). Interestingly, the distance between the observed distribution of non-mānuka taxa and the prediction was less than the distance between the observed distribution of mānuka taxa and the prediction (one-sided Wilcoxon test on Jensen-Shannon divergence *p* = 0.004, see Supplementary Table 7 for Jensen-Shannon divergence values). Again, these observations suggest that factors other than chance, such as dispersal from neighbouring plants, influence the distribution of taxa within the microbiomes of each host group, and that the effect of such inter-host dispersal may be more prominent between mānuka phyllosphere microbiomes than between non-mānuka phyllosphere microbiomes.

H3: mānuka and non-mānuka phyllosphere microbiomes are equivalent sources of bacteria.

To test our third hypothesis, we investigated whether there was an equal ratio of preferential host occupancy in taxa that were shared between mānuka and non-mānuka phyllosphere microbiomes (see ‘Methods’ for definition of preferential host occupancy). Overall, we found that the majority of ASVs that are present in at least three out of six microbiomes had a preferential occupancy in mānuka (i.e. partially dispersed taxa were more likely to be absent from non-mānuka phyllosphere microbiomes) (Fig. 3A). Finally, looking at the total number of unique and shared ASVs across different combinations of host species, we noticed that a substantially smaller number of taxa are shared by any combination of non-mānuka host species, compared to any combination of mānuka and non-mānuka host species (Fig. 3C). In other words, the unequal distribution of bacteria across the phyllosphere microbiomes of mānuka and non-mānuka suggests that a larger number of bacteria have dispersed from mānuka to neighbouring non-mānuka plants than from neighbouring non-mānuka plants to mānuka.

### Mānuka phyllosphere communities are dominated by core bacterial taxa

**Fig. 4:**
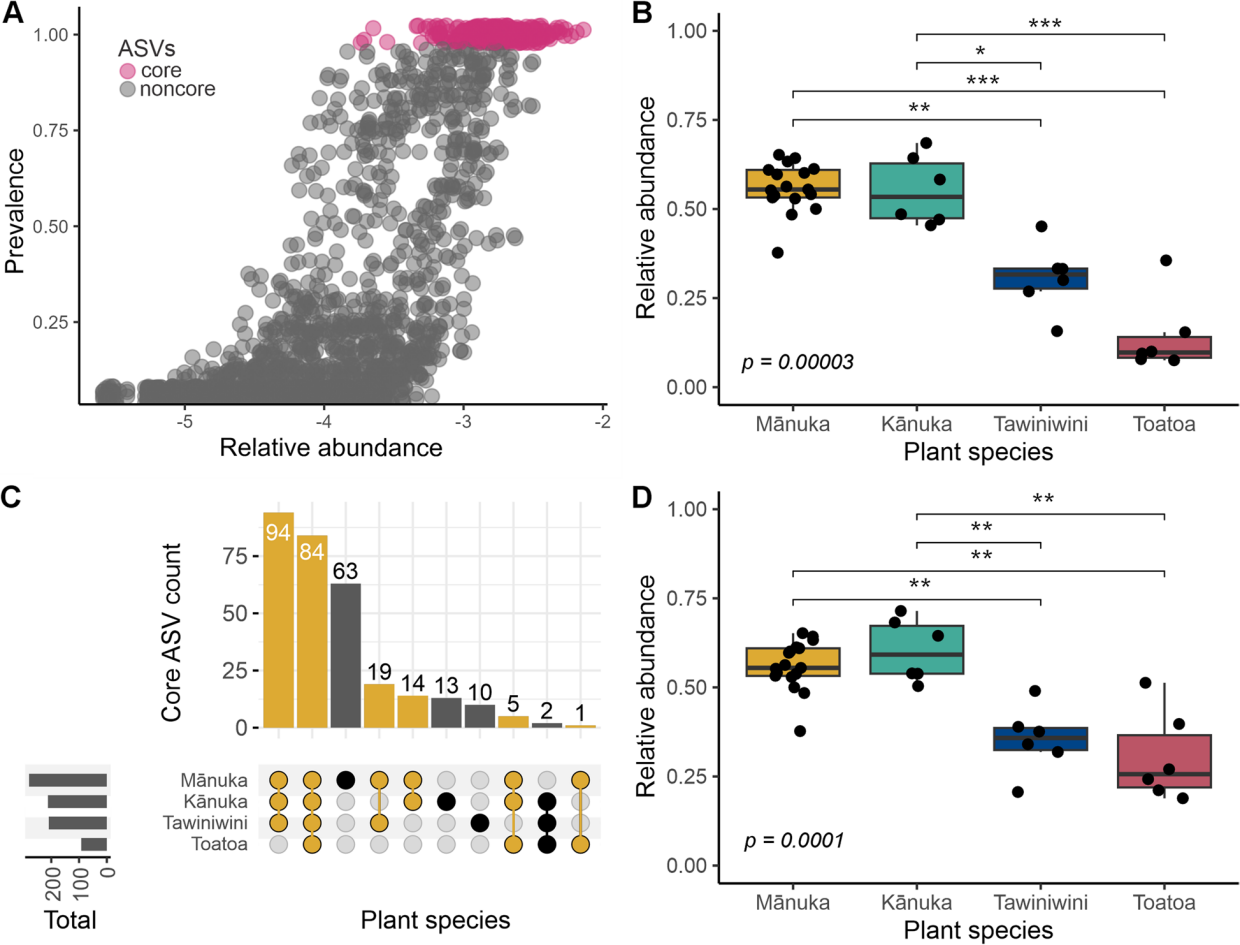
The relative abundance and distribution of host species-specific core microbiomes. **A** Relative abundance (log10) versus prevalence of ASVs in the mānuka phyllosphere microbiome. Pink represents 280 ASVs with 100% prevalence across all mānuka samples that were defined as members of the mānuka core microbiome. **B** The cumulative relative abundance of species-specific core ASVs identified in each individual host species. **C** Upset plot showing the distribution of core ASVs among host plant species. Yellow bars represent host species combinations that include mānuka. **D** The cumulative relative abundance of mānuka core ASVs in each host species. Asterisks indicate significance level of Kruskal–Wallis test

In our previous study, we identified a widely distributed core microbiome in the mānuka phyllosphere [53]. We sought to verify this finding by searching for a core microbiome within our regional and spatially structured study design. Given the importance of defining ecologically relevant core microbiomes within tissue- and species-specific host microbiomes, we used a stringent criterion of 100% prevalence within each individual host species. Using this criterion, we identified 280 core ASVs in the mānuka phyllosphere microbiome (Fig. 4A). These taxa comprised two phyla (Acidobacteria and Proteobacteria) and four genera (*Granulicella* and *Terriglobus*, as well as *1174–901-12* (Rhizobiales) and *Methylocella*, respectively) (Supplementary Fig. 4A). We also applied this core criterion to each neighbouring plant species. Across all kānuka samples, 212 core ASVs were identified (Supplementary Fig. 5A). These taxa comprised two phyla (Acidobacteria and Proteobacteria) and three genera (*Granulicella*, as well as *1174–901-12* and *Methylocella*, respectively) (Supplementary Fig. 4B). Across all tawiniwini samples, 209 ASVs were identified (Supplementary Fig. 5B). These taxa comprised two phyla (Acidobacteria and Proteobacteria) and three genera (*Granulicella*, as well as *1174–901-12* and *Methylocella*, respectively) (Supplementary Fig. 4C). Lastly, 92 core ASVs were identified across all toatoa samples (Supplementary Fig. 5C). These taxa comprised a single phylum (Proteobacteria) and two genera (*1174–901-12* and *Methylocella*) (Supplementary Fig. 4D). The relative abundance of core taxa was significantly different for each host species; the core taxa in mānuka and kānuka had a greater relative abundance compared to core taxa in tawiniwini and toatoa (Benjamini–Hochberg corrected Kruskal–Wallis *p* < 0.05, Fig. 4B).

We next examined the overlap of core taxa across host species (Fig. 4C). In total, 63 ASVs in the mānuka core microbiome were unique to mānuka (i.e. not identified in the core microbiome of any other surrounding plant species). In contrast, very few ASVs in the core microbiome of neighbouring host plants were unique to these species as the majority of ASVs identified in the kānuka, tawiniwini, and toatoa core microbiomes were also identified as members of the mānuka core microbiome. The largest proportion of shared core taxa included 94 ASVs that were common to mānuka, kānuka, and tawiniwini. This was followed by 84 ASVs that were identified in the core microbiome of all four host species. Notably, the relative abundance of mānuka core taxa was significantly higher in mānuka and kānuka compared to tawiniwini and toatoa (Benjamini–Hochberg corrected Kruskal–Wallis *p* < 0.05, Fig. 4D).

### Phyllosphere community composition varies across host species

**Fig. 5:**
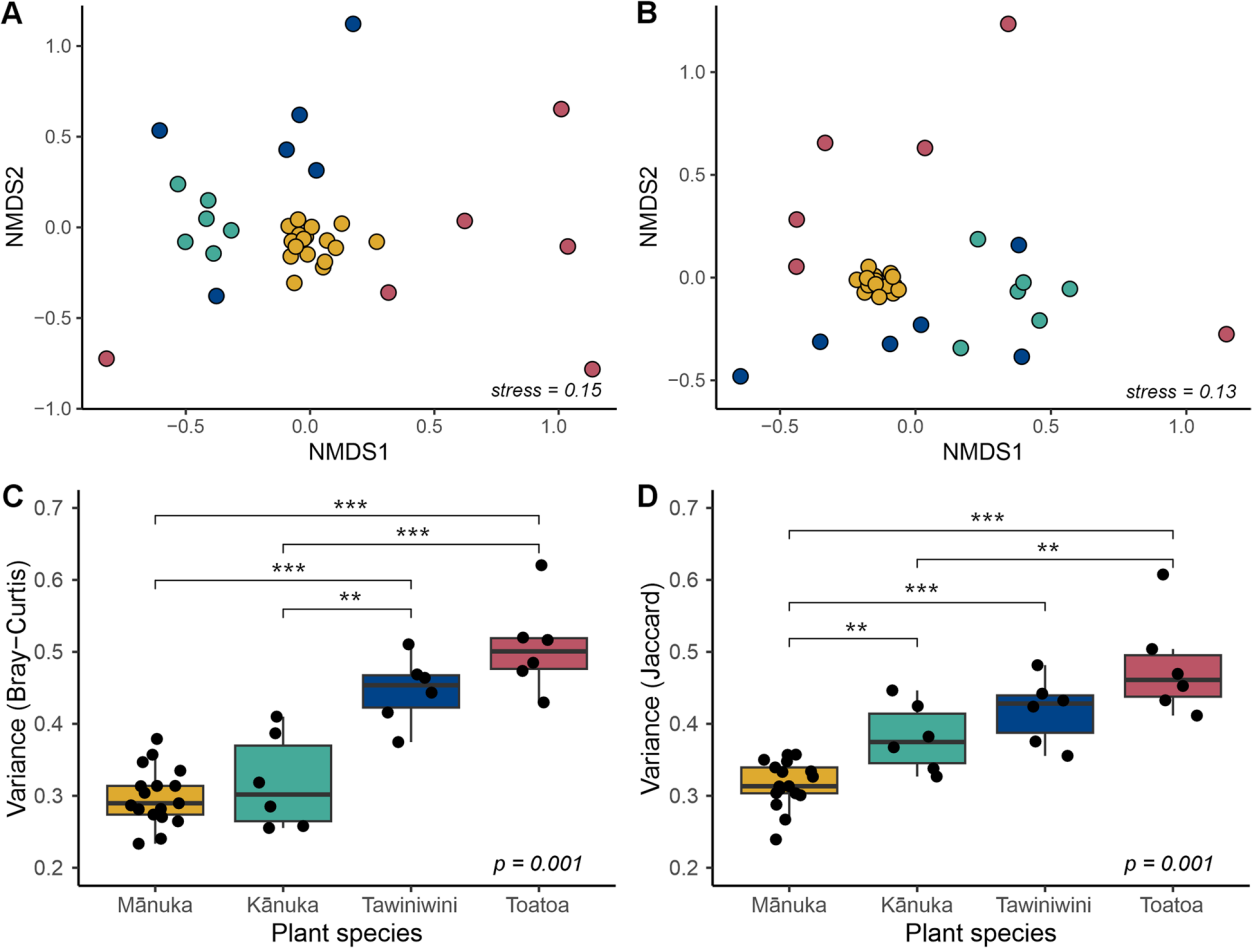
Phyllosphere microbiome community structure shows significant differences across host species. NMDS ordination of **A** Bray–Curtis and **B** Jaccard community dissimilarities (PERMANOVA, *R*^2^ = 0.26, *p* = 0.001). Box plot showing the corresponding intraspecies variance in **C** Bray–Curtis and **D** Jaccard phyllosphere community dissimilarity. Colours depict host species identity. *p* values and asterisks denote significance values of overall and pairwise Betadisper tests, respectively

To investigate the role of host selection in shaping the phyllosphere microbiome, we examined the effect of host species identity on community structure. Across all phyllosphere samples, 16 phyla were detected of which Proteobacteria was the most represented phylum (average relative abundance 61.4%), followed by Acidobacteriota (23.7%), Verrucomicrobiota (5.5%), and Bacteroidota (4.4%) (Supplementary Table 8). Host species-associated differences in composition were detectable at low levels of taxonomic resolution, with six out of the total 16 phyla exhibiting significant differences in relative abundance (Benjamini–Hochberg corrected Kruskal–Wallis *p* < 0.05, Supplementary Tables 9–10). At the ASV level, bacterial community composition was significantly different across host species (Fig. 5A–B) (ANOSIM based on Bray–Curtis and Jaccard; *R* = 0.75, *p* = 0.001 and *R* = 0.77, *p* = 0.001, respectively). Host species identity explained 26% of variation in overall phyllosphere community structure (PERMANOVA on Bray–Curtis and Jaccard dissimilarities, *p* = 0.001). Notably, no significant difference in phyllosphere richness or diversity was identified across host species (*p* > 0.05) (Supplementary Fig. 6A–B, see Supplementary Tables 11–12 for raw and averaged alpha diversity values).

**Fig. 6:**
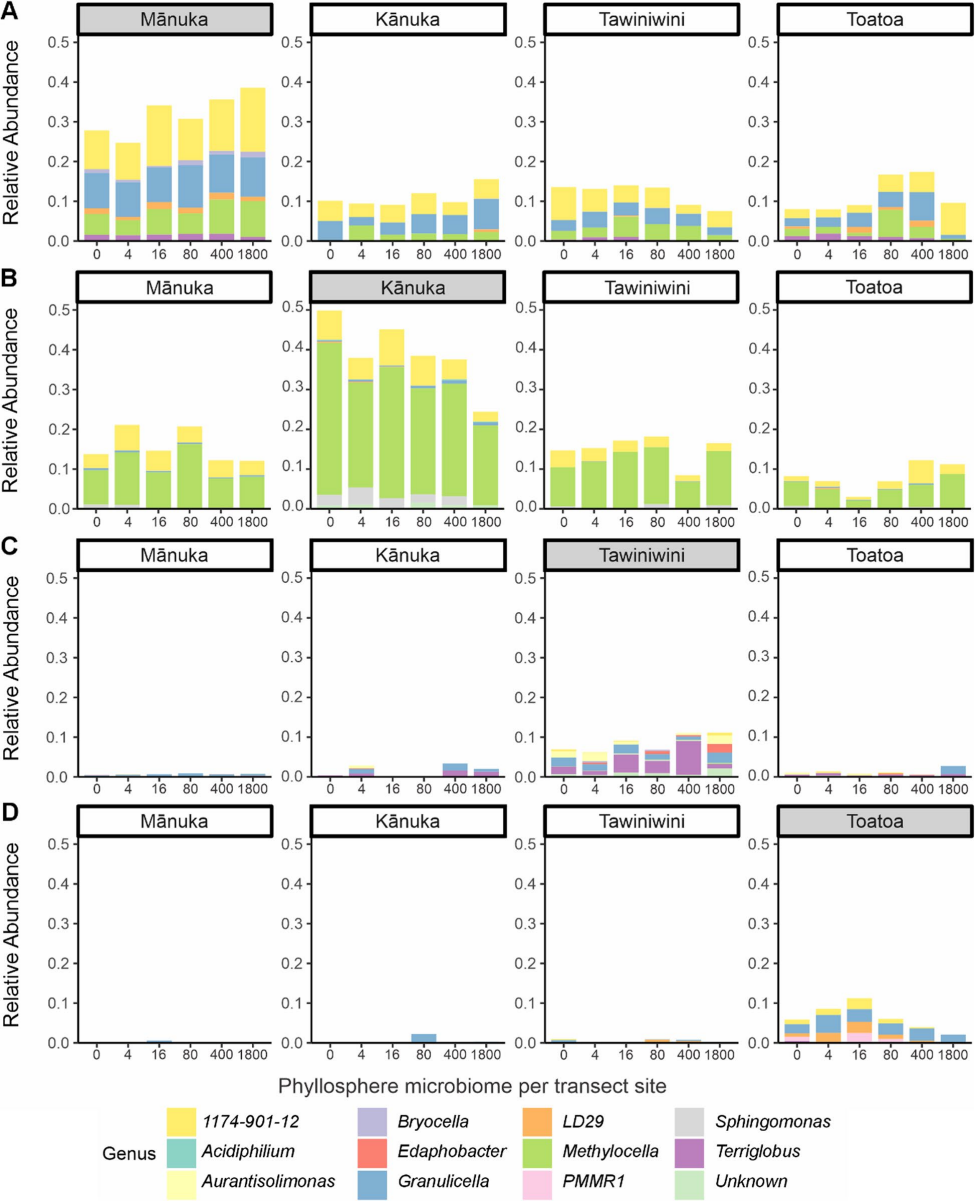
Relative abundance of indicator taxa that are significantly associated with individual host species. Bar plots show the relative abundance of **A** mānuka, **B** kānuka, **C** tawiniwini, and **D** toatoa indicator taxa across all species. Individual bars represent phyllosphere community composition of each plant species at each transect site (i.e. at 0, 16, 80, 400, and 1800 m). Colours represent taxonomic classification at the genus level

To test for significant associations between individual ASVs and each host species, an indicator species analysis was performed and 485 taxa were identified. In total, 217 ASVs were significantly associated with mānuka (*p* < 0.05) and comprised 31.8 ± 5% of the total community in the mānuka phyllosphere. A total of 129 ASVs were significantly associated with kānuka (*p* < 0.05) and comprised 38.9 ± 8.6% of the total community in the kānuka phyllosphere. Only 65 and 74 ASVs were significantly associated with tawiniwini and toatoa (*p* < 0.05), representing 8.5 ± 2.2% and 6.3 ± 3.2% of each host species’ total community, respectively. Indicator taxa were distinctive at the genus level across each of the four host species (Fig. 6A–D). Indicator taxa in the mānuka phyllosphere largely comprised taxa belonging to the genera: *Bryocella*, *Granulicellam*, *LD29*, *Methylocella*, *Terriglobus*, and *1174–901-12*. Meanwhile, indicator taxa in the kānuka phyllosphere largely belong to the genera: *Methylocella*, *Sphingomonas*, and *1174–901-12*. In contrast, indicator taxa in the tawiniwini and toatoa phyllosphere comprised small relative abundances of many of the genera identified in mānuka and kānuka, as well as *Aurantisolimonas* and *Edaphobacter* and *PMMR1*, respectively. Taxonomic differences also persisted at the phylum level (Kruskal–Wallis *p* < 0.05, Supplementary Tables 13–14). Notably, mānuka indicator taxa were present at low relative abundances in neighbouring host species (Fig. 6A). A similar pattern was also observed for kānuka indicator taxa (Fig. 6B). However, indicator taxa identified for toatoa and tawiniwini were essentially absent from the mānuka and kānuka phyllosphere microbiomes (Fig. 6C–D).

### Relative contributions of phyllosphere community assembly mechanisms differ across host species

We used intraspecies variation of phyllosphere community dissimilarity as an indicator of the relative strength of host selection on community structure. Notably, intraspecies variation differed significantly across each of the four host species. Mānuka and kānuka both exhibited significantly less relative abundance-based variation compared to tawiniwini and toatoa (Betadisper *p* < 0.001, Fig. 5C, Supplementary Table 15). Furthermore, mānuka also exhibited significantly less presence/absence-based variation compared to all other neighbouring plant species (Betadisper *p* < 0.001, Fig. 5D, Supplementary Table 15). To complement these results, we also applied a null modelling approach using the normalised stochastic ratio (NST) index to quantify stochasticity in taxonomic community structure [75]. An NST > 0.5 indicates community structure is more likely driven by neutral processes, whereby an NST < 0.5 indicates community structure is more likely driven by deterministic processes. On average, phyllosphere communities belonging to all host species had NST values less than 0.5, indicating the phyllosphere microbiome generally assembles in a deterministic manner (Supplementary Table 16). However, the NST values of mānuka and kānuka phyllosphere communities were significantly lower than the NST values of tawiniwini and toatoa phyllosphere communities, indicating a difference in the magnitude of stochasticity between host species (Supplementary Table 17).

### Distance decay in the phyllosphere microbiome

**Fig. 7:**
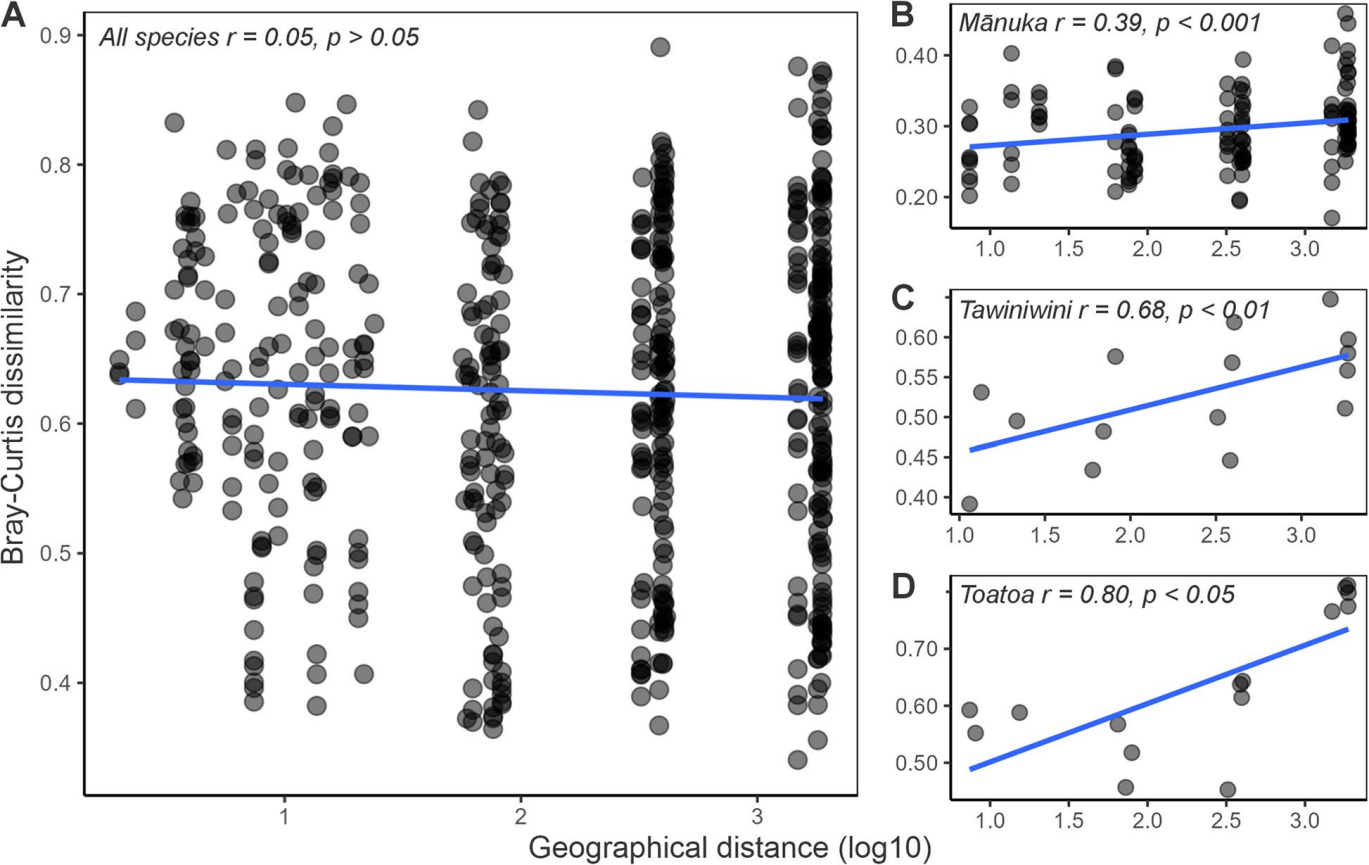
The relationship between phyllosphere community dissimilarity (Bray–Curtis) and geographical distance (log10). **A** No significant distance-decay relationship was observed in the phyllosphere communities across all host species. Significant (*p* values < 0.05) species-specific distance-decay relationships were observed in the high abundance members of the **B** mānuka, **C** tawiniwini, and **D** toatoa phyllosphere microbiomes. Inset *r* values represent Pearson’s product-moment correlation coefficient for each distance-decay

To better understand the role of dispersal limitation in the phyllosphere microbiome, we next used mantel tests to investigate the relationship between community dissimilarity and distance. No overall relationship was observed between phyllosphere community dissimilarity and distance (i.e. across heterospecific samples), highlighting the overarching presence of host species identity (and thus significant interspecies variation) irrespective of spatial distance (Fig. 7A, Supplementary Table 18). We then tested the relationship between community dissimilarity and distance separately for each host species and several significant host-specific distance-decay relationships were identified. Community dissimilarity in the mānuka phyllosphere exhibited a significant relationship with distance (*r* = 0.32, *p* = 0.009). This relationship was observed in low relative abundance community members (*r* = 0.45, *p* = 0.001), high relative abundance members (*r* = 0.39, *p* = 0.001), and taxon presence-absence (*r* = 0.41, *p* = 0.001) (Fig. 7B, Supplementary Table 19). A significant relationship was also present in the high abundance members of the toatoa and tawiniwini phyllosphere (*r* = 0.80, *p* = 0.02; *r* = 0.68, *p* = 0.01, respectively) (Fig. 7C–D, Supplementary Table 19). Similar trends were observed in the kānuka phyllosphere; however, none were statistically significant (Supplementary Table 19). Further, no relationship was observed between taxon presence-absence, low abundance community members, and distance in the phyllosphere microbiome of toatoa and tawiniwini.

### The surface soil microbiome is distinct from the phyllosphere microbiome

Previous studies have suggested surface soil is a source of bacteria for the phyllosphere microbiome [18]. We collected surface soil to evaluate potential contributions of surface soil bacteria to the phyllosphere of different host species. In total, 24 surface soil samples were collected from the base of each sample tree and yielded 15,927 ± 3241 quality-assured reads per sample. In total, 8781 ASVs were identified, with an average of 550 ± 119 ASVs per sample. A total of 25 phyla were detected across all samples (Supplementary Table 20). Proteobacteria were the most represented phyla (average relative abundance 34.2%), followed by Bacteroidota (17.9%), Verrucomicrobiota (17.8%), Acidobacteriota (15.2%), and Planctomycetota (6.0%) (Supplementary Fig. 7). We examined whether host species identity of the tree from which each surface soil sample was collected had any influence on community structure; however, no effect was observed (PERMANOVA on Bray–Curtis and Jaccard dissimilarities *p* > 0.05, Supplementary Fig. 8). Mantel tests were used to test for a decay of community similarity with distance; however, no significant relationship was observed (*p* > 0.05) (Supplementary Table 21). Notably, in contrast to the phyllosphere microbiome, no ASVs were 100% prevalent across surface soil samples (Supplementary Fig. 5D).

Overall, the community structure of surface soil was distinct from the phyllosphere (Supplementary Fig. 9A–B). A significant difference in community richness (*p* = 0.04) but not diversity (*p* > 0.05) was identified between surface soil and phyllosphere microbiomes (Supplementary Fig. 6C–D, see Supplementary Table 22 for raw values). Notably, only 38 ASVs were shared between all phyllosphere microbiomes and surface soil samples (Supplementary Fig. 10A). The number of taxa shared between the phyllosphere microbiomes and surface soil samples was relatively consistent across host species, ranging from 22 to 29 ASVs (Supplementary Fig. 10B–E).

## Discussion

Stochastic processes, such as dispersal, operate in conjunction with host selection to shape the composition of host-associated microbiomes. However, the relative importance of each of these processes in the assembly of the phyllosphere microbiome remains unclear. In our previous study, we identified a core microbiome in the *Leptospermum scoparium* (mānuka) phyllosphere that was persistent across five environmentally diverse and geographically distinct populations [53], making the mānuka phyllosphere an ideal model system for interrogating processes of community assembly. In this study, we used a multi-species, spatially explicit sampling design to examine the relative influence of host species identity and spatial distance in the assembly of the mānuka phyllosphere microbiome. Overall, host species identity had a stronger influence on phyllosphere community composition than distance. However, the relative influence of host species identity was different for each host species. Compared to ecologically and morphologically similar plant neighbours, a consistent and distinct community composition was observed in the mānuka phyllosphere microbiome, providing strong evidence in support of our hypothesis that host selection is the main driver of community assembly on the mānuka leaf surface. Conversely, the phyllosphere microbiome of kānuka (*Kunzea ericoides*), tawiniwini (*Gaultheria antipoda*), and toatoa (*Phyllocladus alpinus*) exhibited a higher degree of compositional stochasticity. Accordingly, the phyllosphere microbiome of non-mānuka native plants tended to exhibit stronger distance-decay relationships than the phyllosphere microbiome of mānuka. Furthermore, the distribution of taxa within each site deviated significantly from random probability distributions, suggesting that the local movement of bacteria among phyllosphere microbiomes of adjacent plant species is not purely random.

Intraspecies variation in phyllosphere community composition was significantly different across the four host species in our study, suggesting that the capacity to select for a consistent phyllosphere microbiome, and thus the relative strength of host selection, is variable across plant species. Previous studies have reported contradictory results regarding the relative importance of host selection versus microbial dispersal in the assembly of the phyllosphere microbiome. For example, Redford et al. [24] found that the intraspecies variation of phyllosphere communities on *Pinus ponderosa* trees separated by distances of up to 14,000 km was significantly less than the interspecies variation between *P. ponderosa* and sympatric *Pinus* species. In contrast, Finkel et al. [28] reported that the interspecies variation between co-occurring *Tamarix* species was less than the intraspecies variation of allopatric *Tamarix* species. However, in light of our findings, it is plausible that plant species vary strongly in the degree to which they associate and interact with their phyllosphere microorganisms. Hence, we propose that host selection of phyllosphere communities is an essential hypothesis that requires empirical testing on a species-by-species basis before embarking on experimental studies. Identifying plant species that exert strong selection on their phyllosphere microbiome—achieved empirically through the identification of strong host association patterns—will be paramount for generating targeted and falsifiable hypotheses designed to enhance our understanding of specific and potentially beneficial plant-microbial relationships in the natural phyllosphere. These hypotheses can then be tested using rational experimental designs, which have historically founded our understanding of plant-pathogen interactions in the phyllosphere [76].

Despite the growing number of empirical-based studies that demonstrate patterns of host species identity in phyllosphere community composition [16, 20, 26, 27, 77–79], the mechanism(s) of host selection in the phyllosphere microbiome are still mostly elusive. Our unique comparison of phyllosphere communities belonging to plant species that exhibit ecological and morphological similarity to one another permits mechanistic interpretation. We noticed that several general characteristics of the kānuka phyllosphere microbiome, such as intraspecies variation (Fig. 5C–D) and the relative abundance of core (Fig. 4B and D) and indicator (Fig. 6) taxa, were more similar to mānuka than either of the other two plant species. Considering that mānuka and kānuka are morphologically similar species, this may be a result of similar selection pressures exhibited by the shared characteristics of their leaf surfaces. For example, mānuka and kānuka trees in our study region have similar leaf nitrogen, stomatal conductance, specific leaf area, and wood density [80, 81], traits that have been previously associated with interspecies variation in both bacterial and fungal phyllosphere community composition [27, 77, 82]. However, despite these similarities, the composition of the mānuka phyllosphere microbiome remained distinct from that of kānuka, suggesting that the mechanism of selection in the mānuka phyllosphere microbiome is independent of these general leaf traits. This observation, together with our previous study that identified a remarkably persistent host association [53], is parsimonious with mānuka having a direct influence on the structure and composition of its phyllosphere bacterial communities. Interestingly, mānuka is a highly aromatic plant species and mānuka leaves exhibit distinct chemical profiles compared to kānuka leaves. For example, the volatile organic compounds (VOCs) in mānuka leaves are comprised of monoterpene hydrocarbons (5%), sesquiterpene hydrocarbons (60–70%), and triketones (20%) [83]. In contrast, the VOCs in kānuka leaves are largely comprised of monoterpenes (75%) [84]. Therefore, it is conceivable that a chemically mediated mechanism of host selection may be taking place in the mānuka phyllosphere microbiome. The direct recruitment of microorganisms via the secretion of specific chemical molecules, including plant-derived VOCs, has been well documented in the rhizosphere and endosphere [85, 86]. Furthermore, direct recruitment of a disease-suppressive microbiome via specific chemical-signalling has been recently demonstrated in the phyllosphere of tomato [5].

Compared to mānuka, the community structure of the toatoa and tawiniwini phyllosphere microbiome appeared highly variable (Fig. 5A–B), suggesting that stochastic processes play a larger role than the identity of the host in shaping the phyllosphere microbiome of these plant neighbours. In accordance with this observation, theoretical studies have shown that stochastic processes are more likely to contribute to microbiome community structure when selective forces by the host are considerably reduced [38]. Further, abundant taxa in the toatoa and tawiniwini phyllosphere microbiome exhibited a strong and significant decay in community similarity with distance (Fig. 7C–D), highlighting a role of dispersal limitation in structuring the abundant members of their communities. A significant, albeit weaker, distance-decay relationship was also identified in mānuka phyllosphere communities (Fig. 7B), which also demonstrates the underlying role of stochastic processes in shaping the structure of the phyllosphere communities even in the presence of strong host selection. This finding is in-line with a previous greenhouse study that found spatial variation in the relative abundance of phyllosphere taxa on *Arabidopsis thaliana*, despite a strong convergence in communities over time [34]. It is notable that we only observed distance-decay relationships within individual host species, rather than across different host species (Fig. 7). This shows us that even though the assembly of the tawiniwini and toatoa phyllosphere microbiome was more stochastic than mānuka, the effect of dispersal in the presented study was not strong enough to obscure the overall effect of host species identity (i.e. intraspecies variation was on average smaller than interspecies variation). The identification of species-specific distance-decay relationships supports two previous studies that observed significant relationships within the phyllosphere of a single host species [29, 40], and may also explain why distance-decay relationships have generally not been observed across heterospecific phyllosphere samples [20]. Moreover, these findings emphasise the importance of choosing appropriate sampling scales when testing for specific ecological patterns, such as using exponentially increasing distances for testing exponential models of distance-decay [36].

While mānuka’s phyllosphere community was distinct, we did observe an overlap of abundant taxa across host species. Within our sampling design, focal mānuka and neighbouring plant species were separated by close and consistent distances (~ 4 m) to equalise dispersal opportunities among host species. However, the distribution of bacteria within each site was not congruent with a scenario in which taxa were dispersed with equal probability across mānuka and neighbouring phyllosphere microbiomes (Fig. 3A–B). Instead, a large proportion of taxa was shared between mānuka and non-mānuka host species while few taxa were shared among non-mānuka host species (Fig. 3C). Further, taxa that were shared among multiple microbiomes typically had a higher occurrence in mānuka than neighbouring host species (Fig. 3A). While speculative, a logical interpretation of these results is that inter-host dispersal may be occurring in such a way that mānuka is acting as a source of specific phyllosphere microorganisms for neighbouring plant species. Other evidence in support of this idea includes the presence of key members of the mānuka microbiome (i.e. core ASVs and indicator taxa) in neighbouring species and a correspondent lack of neighbouring plant species’ key taxa in mānuka. Furthermore, this interpretation is in agreement with three previous lines of evidence: (1) plants can influence the composition of local airborne bacterial communities [87], (2) the presence of plant neighbours is linked to variation in phyllosphere bacterial community size and composition (i.e. neighbour effect) [41, 88], and (3) neighbour effect varies with crop plant species identity [41, 88]. Our study builds on these results by suggesting a source-sink dynamic may establish between phyllosphere microbiomes that experience different relative strengths of host selection. An alternative explanation could be that shared taxa between mānuka and neighbouring plant species are cosmopolitan within our sampling region. However, this interpretation is less congruent with the specificity of the mānuka phyllosphere. In addition, phyllosphere taxa were essentially absent in surface soil, confirming the distinctiveness of the phyllosphere microbiome from the surrounding environment. Together, our results raise the question as to whether conventional sampling designs used to infer the neutral role of dispersal are sufficient to capture the complex reciprocal nature of dispersal in the phyllosphere microbiome [36, 37]. Incredibly strategic sampling designs will be required to shed further light on these complicated and intricately linked processes.

Our work demonstrates the potential of mānuka as an intriguing model plant species for developing our mechanistic understanding of plant-microbiome interactions in the phyllosphere of woody perennials. Now that we have established that a strong, species-specific association (and thus host-microbiome interaction) likely exists between mānuka and its associated phyllosphere microorganisms, using metagenomic analyses to target mānuka-specific metagenome-assembled genomes will be a valuable next step to identify microbial functions that are specifically associated with the identity of the mānuka host. Cultivation approaches, manipulative experiments, and microscopy will also be essential to elucidate the functional potential of target microorganisms and the influence they have on the functional traits of mānuka, including economically important traits such as nectar DHA. Finally, continued investigation utilising the unique comparison provided by mānuka and kānuka will be beneficial to confirm a role of chemical signalling in host selection of mānuka phyllosphere bacterial communities.

## Conclusions

An ongoing debate exists in the literature regarding the relative importance of host selection versus microbial dispersal in the assembly of natural phyllosphere bacterial communities. Using a systematic sample design in a native New Zealand bush, we demonstrate the first attempt to quantify and compare the contribution of host species identity and dispersal in the phyllosphere microbiome of different plant species. Moreover, our results reveal that the relative influence of each of these processes is not universal across plant species. The mānuka (*L. scoparium*) phyllosphere microbiome appeared to be more strongly influenced by host selection, whereas the phyllosphere microbiome of neighbouring native plant species appeared to be more strongly influenced by microbial dispersal. Furthermore, the distribution of phyllosphere taxa within each site reflects a scenario in which microorganisms disperse between neighbouring host plants (i.e. inter-host dispersal). However, as an extension to this previously established concept, we provide new evidence that suggests that the relative strength of host selection influences inter-host dispersal such that phyllosphere microbiomes that are strongly influenced by host selection may act as a source of microorganisms to phyllosphere microbiomes that are only weakly influenced by host selection. Overall, the evidence presented in this study emphasises the importance of using explicit terminology, carefully structured sampling designs, and falsifiable hypotheses to investigate the complex ecological processes that drive the assembly of phyllosphere bacterial communities in natural environments. Several new perspectives are provided for future investigations focused on advancing our mechanistic understanding of community assembly and plant-microorganism relationships in the phyllosphere.

## Supplementary Information

Supplementary Material 1.

## Data Availability

All raw sequencing data is available on NCBI SRA under the accession number PRJNA1108893 and SRA accessions SRX24489499-SRX24489558. The R scripts used for our main analyses and figures can be found on the GitHub page https://github.com/asn6/NZ-phyllosphere-transect.
