# Supplementary material for: Host selection is not a universal driver of phyllosphere community assembly among ecologically similar native New Zealand plant species
Source: Microbiome. 2025 Jan 31;13:35. doi: 10.1186/s40168-024-02000-x (PMC11786578; doi:10.1186/s40168-024-02000-x)
Supplement: Supplementary file 2 — Supplementary Material 1. [file 40168_2024_2000_MOESM1_ESM.docx]

**Supplementary Figures and Tables**


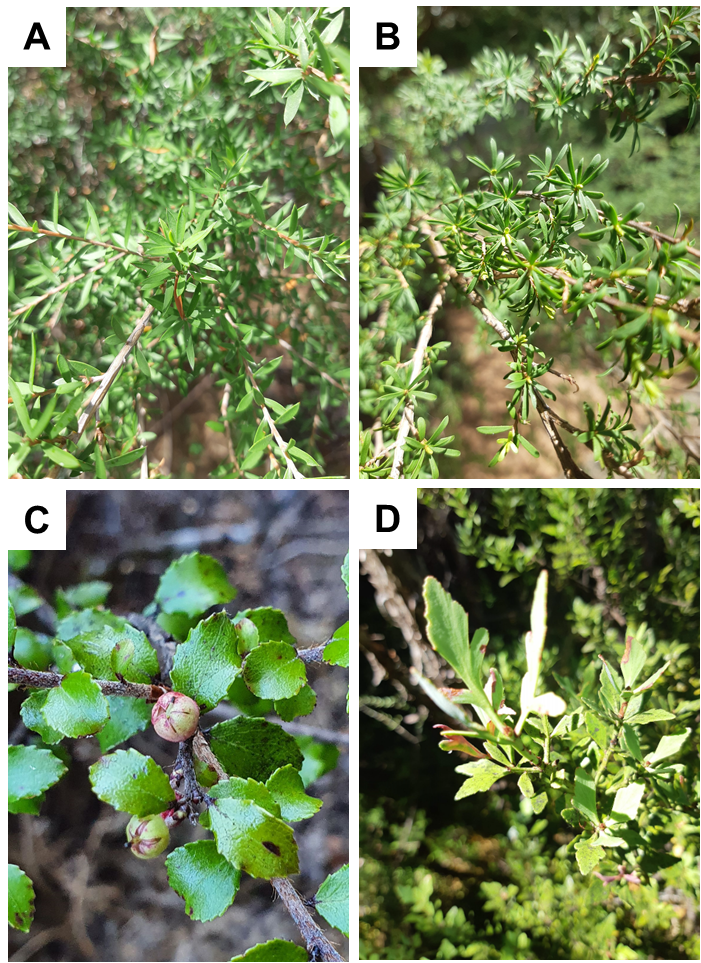


**Supplementary Fig. 1:** Leaf morphologies of sampled mānuka (*Leptospermum scoparium*)(A) and neighbouring plant species kānuka (*Kunzea ericoides*)(B), tawiniwini (*Gaultheria antipoda*)(C), and toatoa (*Phyllocladus alpinus*).


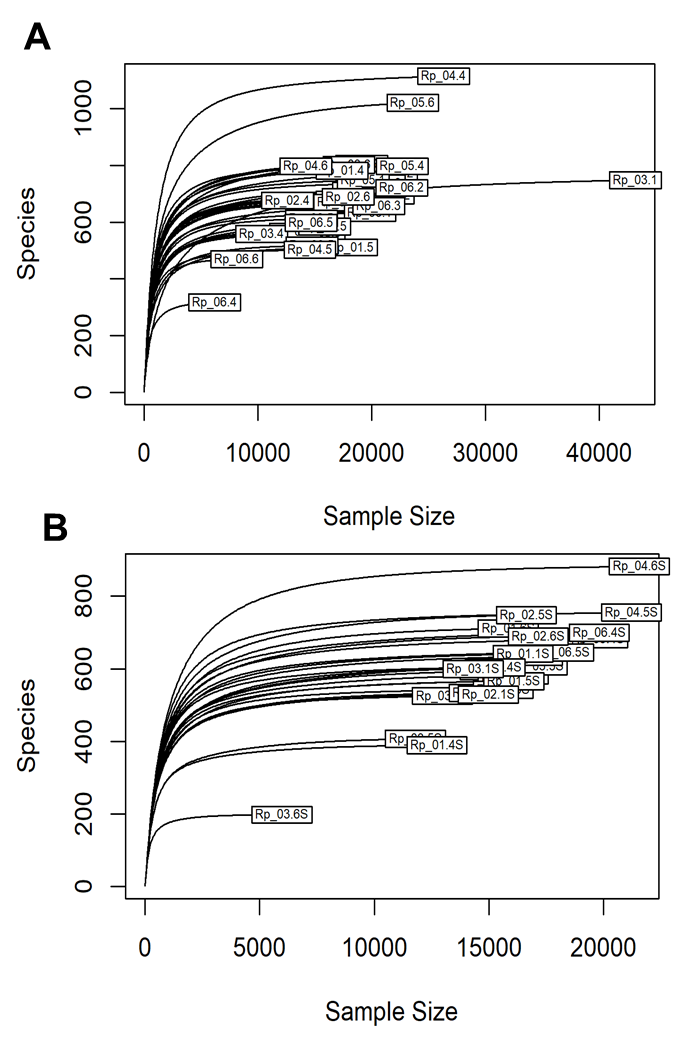


**Supplementary Fig. 2:** Rarefaction curves showing the relationship between the number of ASVs detected and the sample size (i.e. number of sequencing reads) for the phyllosphere (A) and surface soil (B). Rp_03.1 stands out from other phyllosphere samples due to its large number of reads.


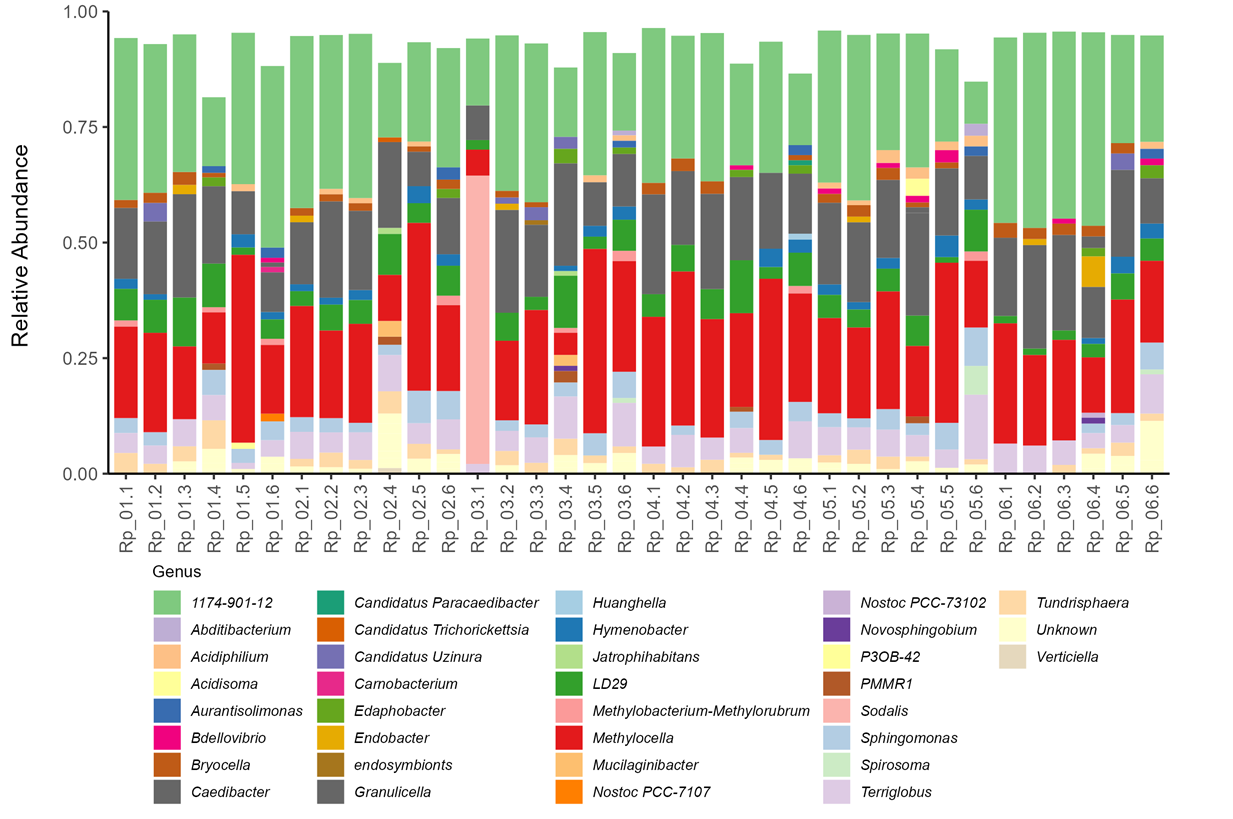


**Supplementary Fig. 3:** Relative abundance of genera (relative abundance > 0.01) in the phyllosphere. Rp_03.1 is dominated by 32 ASVs belonging to the genera *Sodalis*, which comprise 62.4% of reads.**
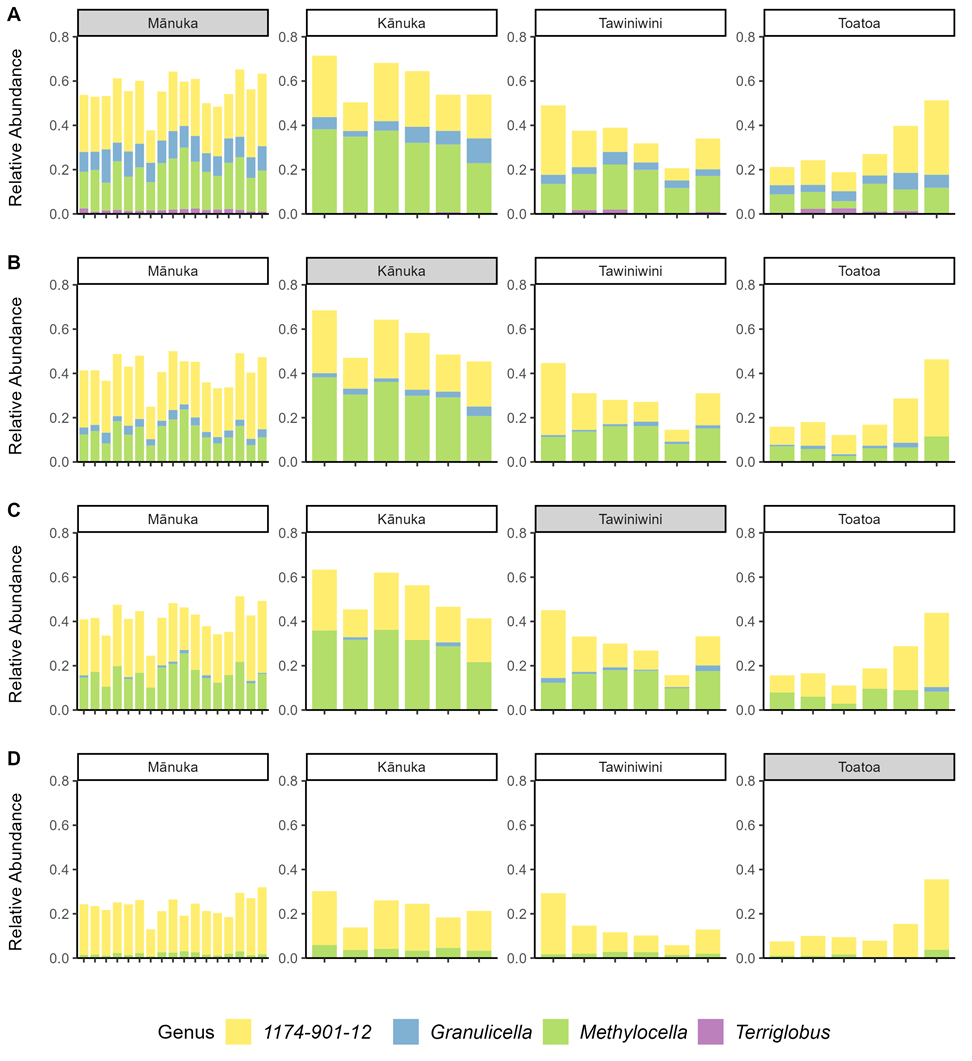
**

**Supplementary Fig. 4:** The relative abundance of core ASVs belonging to each host species: (A) mānuka (280 ASVs), (B) kānuka (212 ASVs), (C) tawininwini (209 ASVs), and (D) toatoa (92 ASVs), across the phyllosphere of each and every host species. Colour represents taxonomic assignment at the genus level.


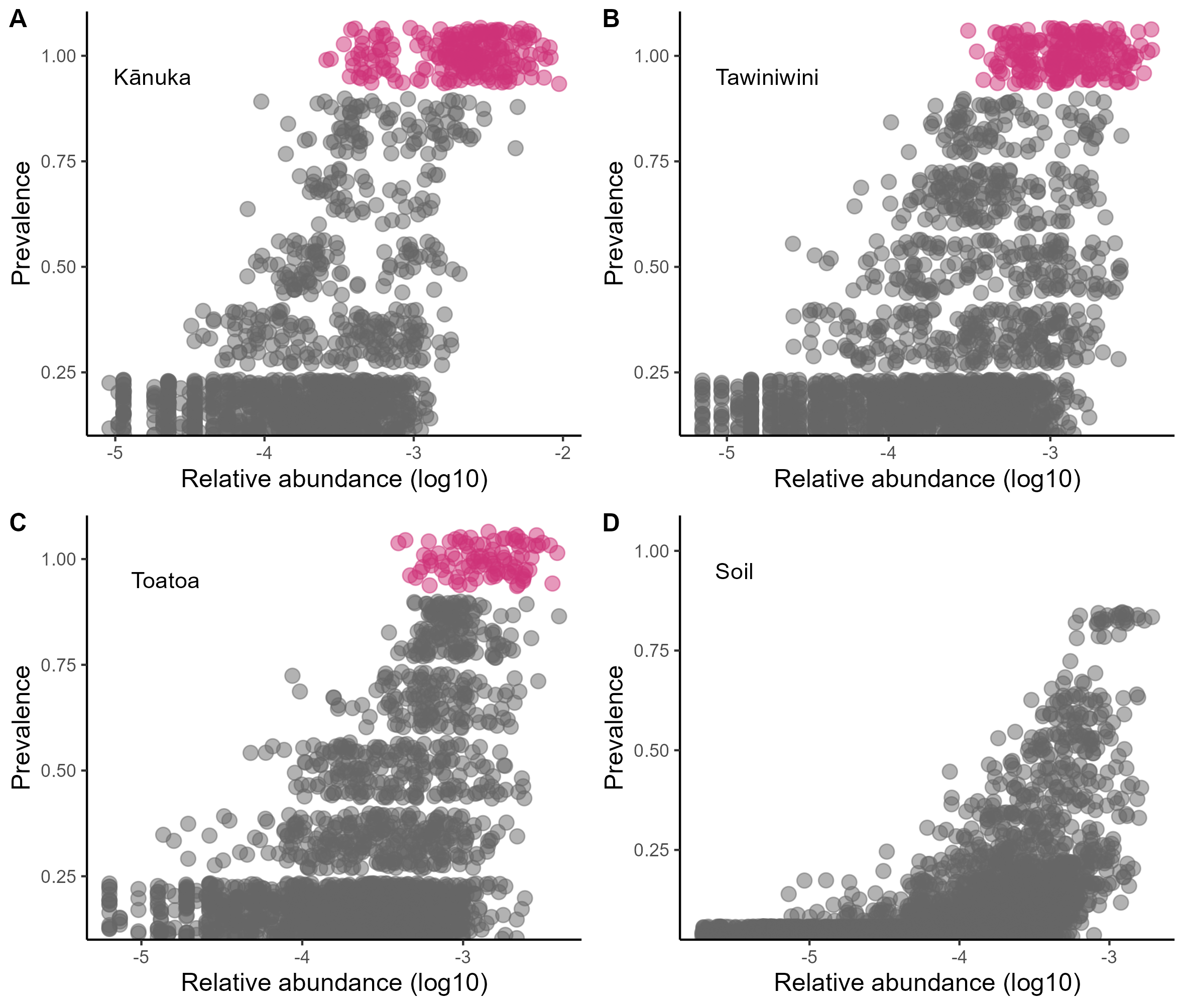


**Supplementary Fig. 5:** Relative abundance (log10) vs. prevalence of taxa in the phyllosphere microbiome of neighbouring host species kānuka (A) tawiniwini (B), toatoa (C) and surface soil (D). Colour indicates core taxa with 100% prevalence in each of these host species.


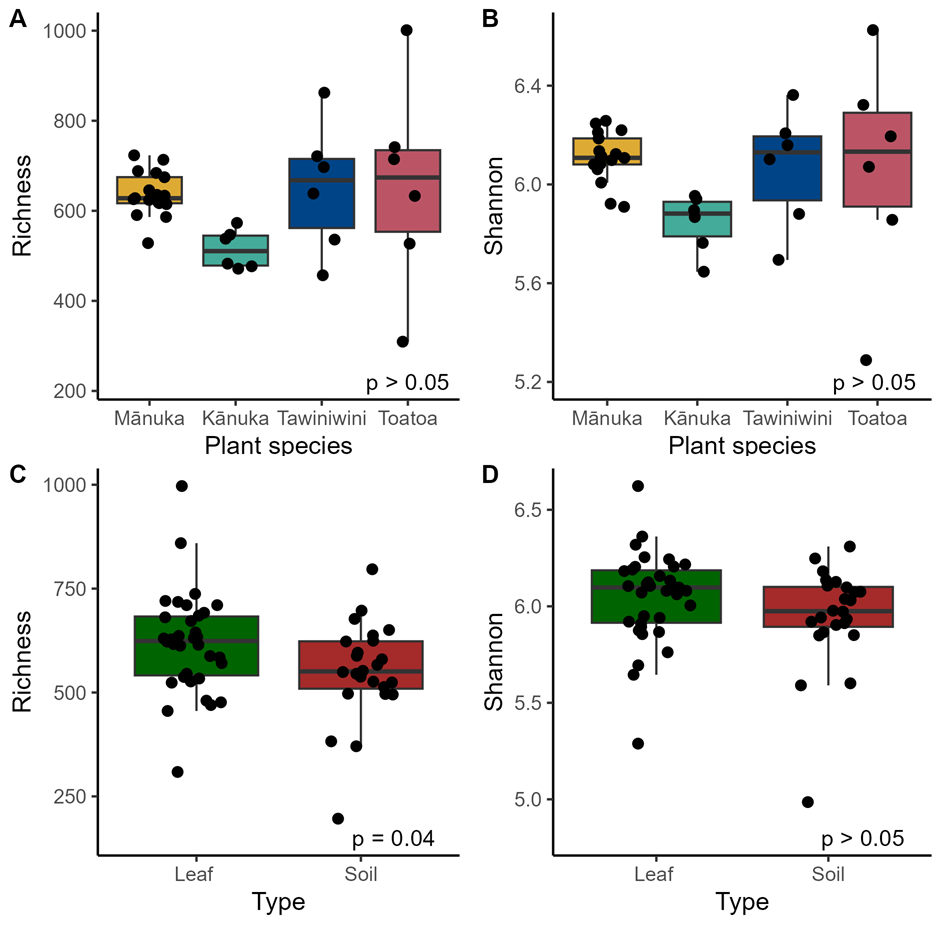


**Supplementary Fig. 6:** Alpha diversity (Shannon and richness) of the phyllosphere microbiome across different host species (A-B) and sample types (C-D). Each sample was subsampled (100x) to an even sequencing depth. Boxes indicate the interquartile range and the thick bar represent the median. Vertical segments extend to the fifth and 95^th^ percentiles of the distribution of values. P values represent significance of ANOVA and Wilcox test for host species and sample type, respectively.

**Supplementary Fig. 7
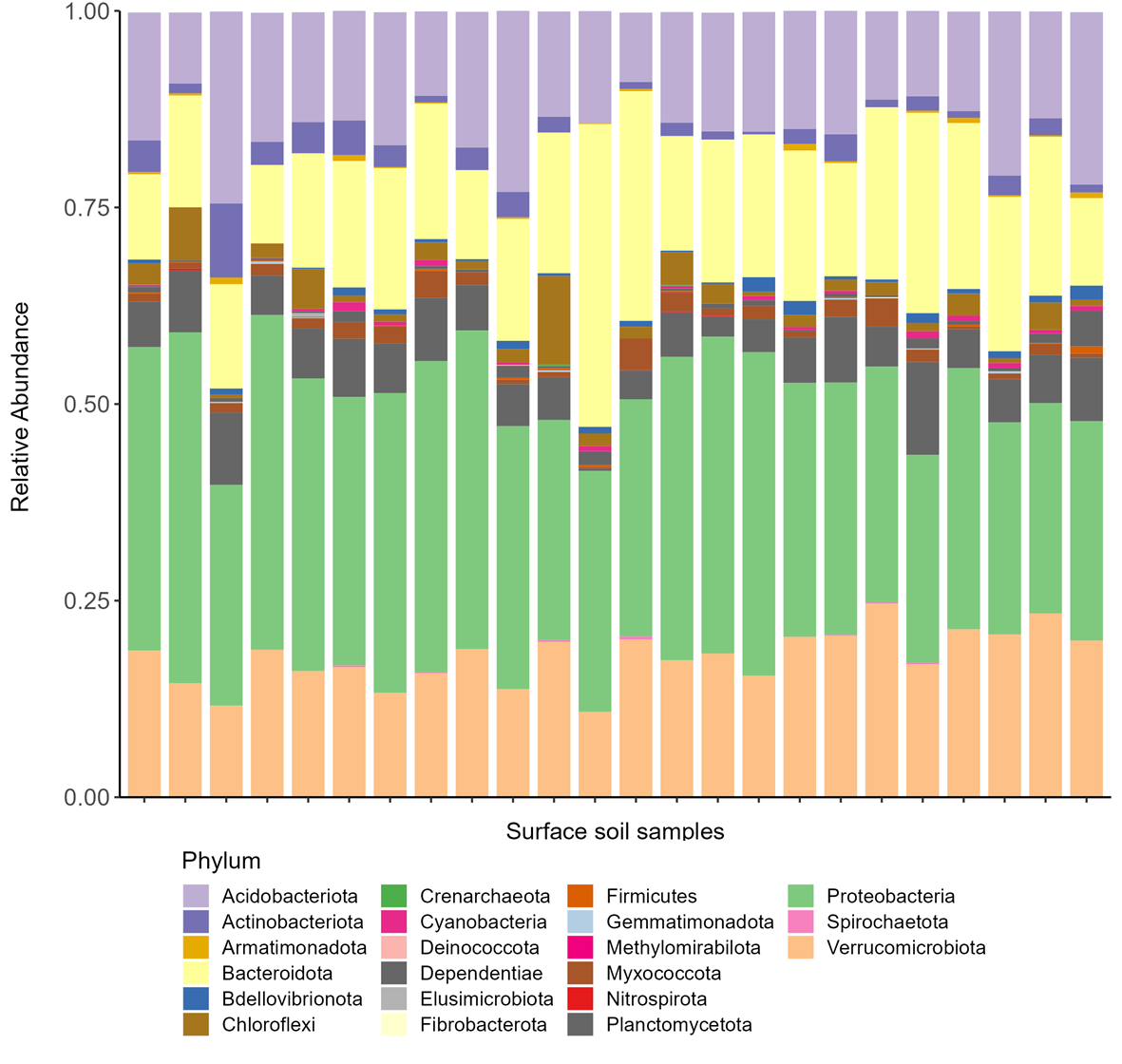
:** Relative abundance of phyla (relative abundance > 0.001) in surface soil. Colour depicts taxonomic assignment at the phylum level.


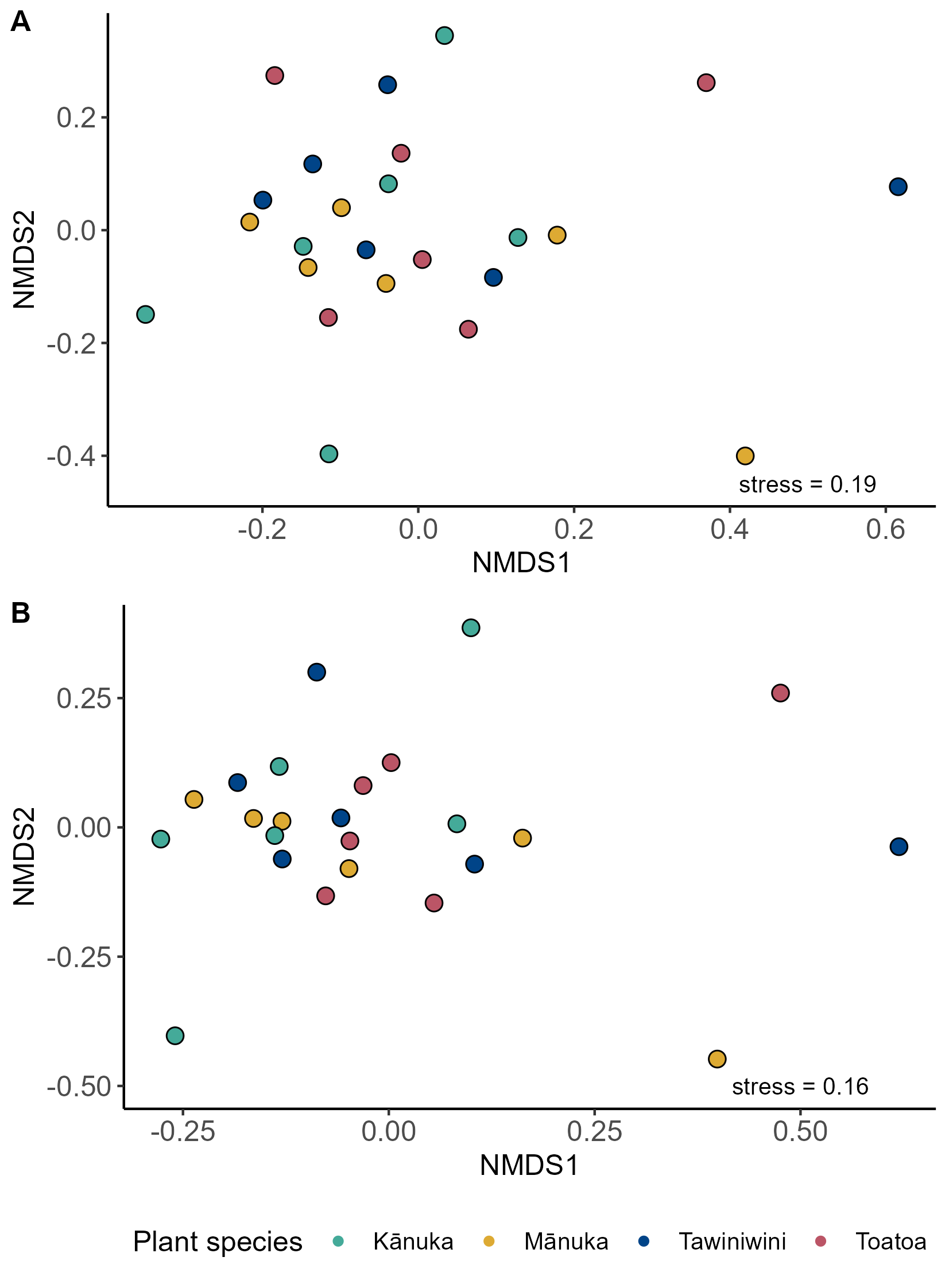


**Supplementary Fig. 8:** NMDS ordination of surface soil microbial community dissimilarity based on A) Bray Curtis (relative abundance) and B) Jaccard (presence/absence).


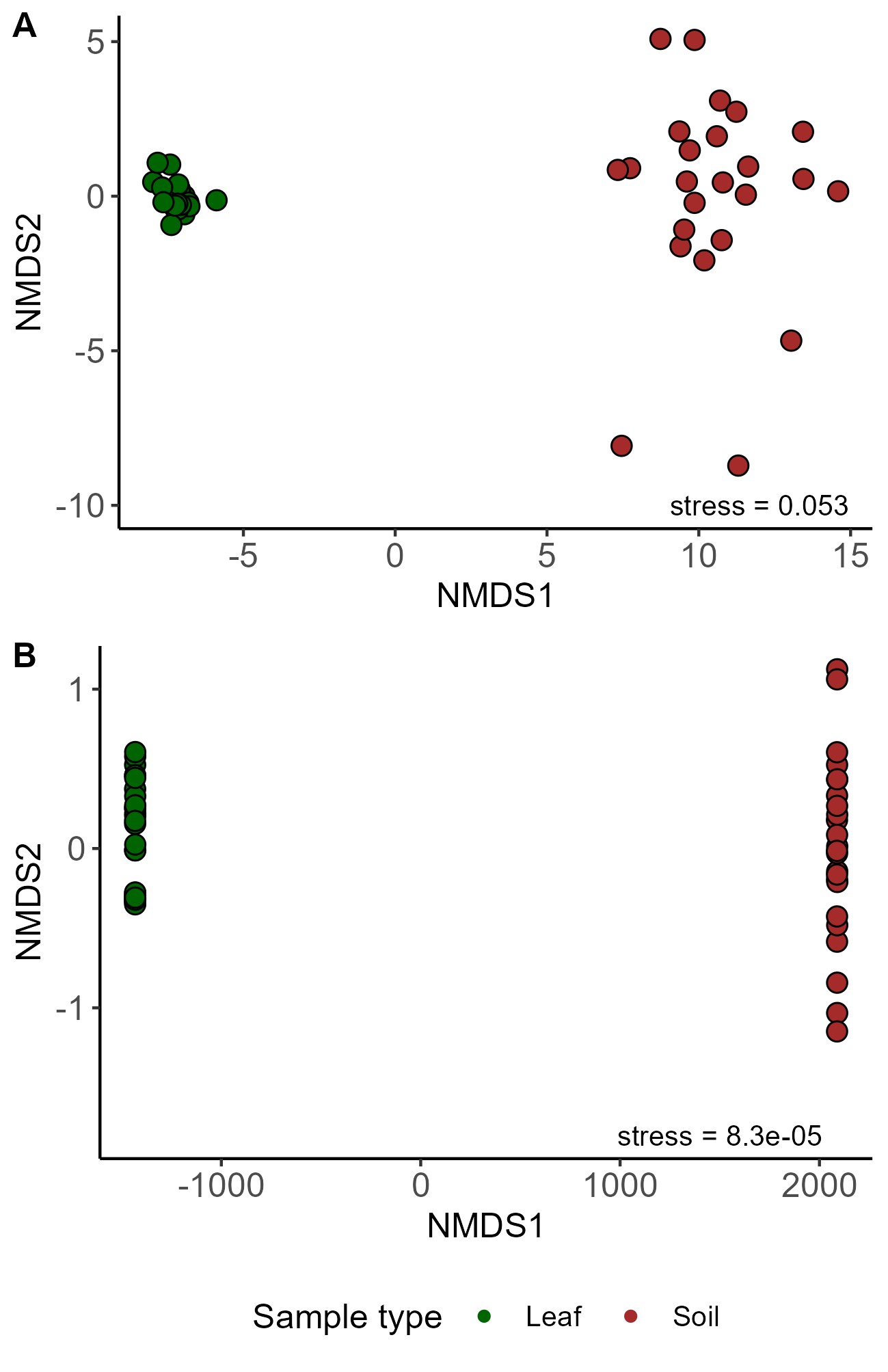


**Supplementary Fig. 9:** NMDS ordination of surface soil and phyllosphere community dissimilarity with A) Bray-Curtis (relative abundance) and B) Jaccard (presence/absence)


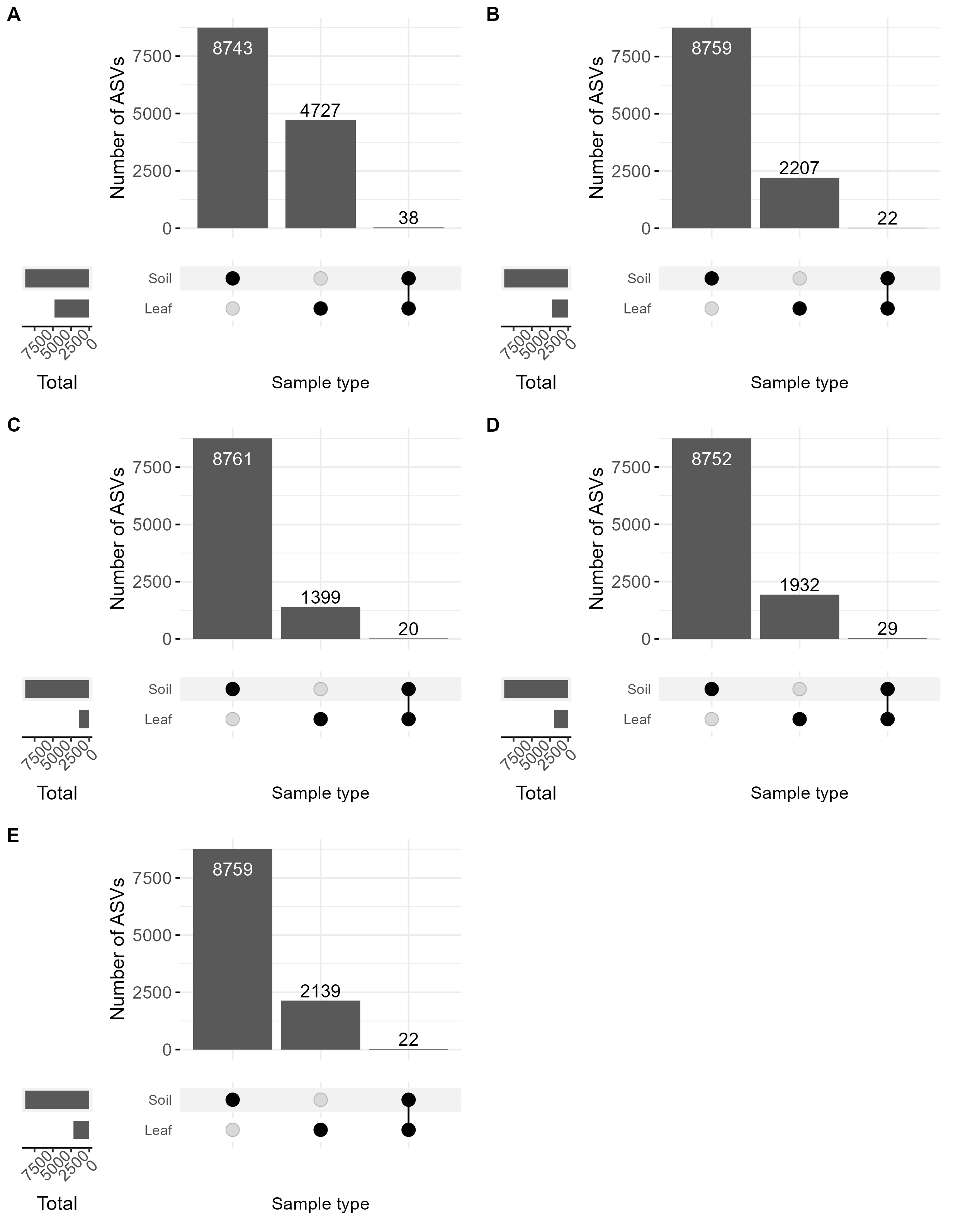


**Supplementary Fig. 10:** The total number of shared and unique ASVs across surface soil and phyllosphere samples for all (A) and independently for mānuka (B), kānuka (C), tawiniwini (D), and toatoa (E) host species.

**Supplementary Table 1:** Descriptions of mānuka (*Leptospermum scoparium*), Kānuka (*Kunzea ericoides*), tawiniwini (*Gaultheria antipoda*), and toatoa (*Phyllocladus alpinus*) species in New Zealand.

|  |  | *Leptospermum scoparium^[[1]](#footnote-1)^* | *Kunzea ericoides^[[2]](#footnote-2)^* | *Gaultheria antipoda^[[3]](#footnote-3)^* | *Phyllocladus alpinus^[[4]](#footnote-4)^* |
| --- | --- | --- | --- | --- | --- |
| General information | Common name | Mānuka | Kānuka | Tawiniwini | Mountian toatoa |
|  | Family | Myrtaceae | Myrtaceae | Ericaceae | Podocarpaceae |
|  | Structural class | Dicotyledon | Dicotyledon | Dicotyledon | Dicotyledon |
|  | Angiosperm/ gymnosperm | Angiosperm | Angiosperm | Angiosperm | Gymnosperm |
|  | Distribution | Indigenous to NZ and Australia.  Coastal to low alpine throughout NZ.  Pioneer species. Commonly found with Kānuka ^[[5]](#footnote-5)^ | Endemic to NZ  Coastal to low alpine throughout North and northern South Islands, NZ.  Pioneer species^[[6]](#footnote-6)^  Commonly found with mānuka. | Endemic to NZ  Lowland to subalpine scrub in the South and central North Islands, NZ | Endemic to NZ  Scrub forests to subalpine throughout NZ, particularly colder climates |
|  | Chromosome number | Normally diploid (22) | Diploid (22) | Diploid (22) | Diploid (18) |
|  | Mature plant height | 2-10 m  (2-5 m in study site) | Up to 20 m  (2-8 m in study site) | 1-2 m | Up to 9 m |
| Leaf characteristics | Foliage | Evergreen | Evergreen | Evergreen | Evergreen |
|  | Leaf size | 4-12 mm long | 4-12 mm long | 5-15 mm long | Phylloclades 5-25 mm long, 3-12 mm wide. |
|  | Specific leaf area | 7.26±0.25 m^2^kg^–1^ ^[[7]](#footnote-7)^ | 7.92±0.39 m^2^kg^–1^ |  |  |
|  | Leaf arrangement | Single or in groups | Single or in groups | Singly along stem | Singly and distichously along stem |
|  | General leaf description | Undivided, linear to ovate, sharply pointed^[[8]](#footnote-8)^  Anomocytic stomata  Thick, striated cuticle (more than 10 µm)  Non-glandular, unicellular, appressed hairs on their young leaves normally shed during leaf development  No papillae | Undivided, narrow-linear, pointed tips | Undivided, round-oblong, leathery, shiny, small serrations | Phylloclades pinnately leaf-like, undivided, of variable shape  Seedlings have needle leaves, but these become ephemeral and non-photosynthetic as plants develop |
| Flower characteristics | Flower size | 8-25 mm diam | 4-8 mm diam | 3-4 mm diam | Pollen cone 4-6 mm long |
|  | General flower description | Solitary, white or pink, 5 petals | Solitary but occur in clusters, white or prink, 5 petals | Solitary, white or pink, bell-shaped, 5 petals | - |
|  | Regional flowering time | February | February | November | - |
|  | Fruit | Dry | Dry | Dry | - |
| Seed mass/size |  | 5-6 mm | 2-3 mm |  |  |
| Wood density |  | 400-600 kg m^3^ ^[[9]](#footnote-9)^ | 400-600 kg m^3^ ^[[10]](#footnote-10)^ |  |  |
|  | Breeding system | Andromonoecious | Hermaphrodite | Hermaphrodite | Hermaphrodite |

**Supplementary Table 2:** ASV occurrence probabilities predicted for each microbiome occurrence

| Microbiome occurrence^*^ | Probability an ASV is found at an occurrence, with zero truncation^+^ |
| --- | --- |
| 0 | NA |
| 1 | 6/63 = ~0.095 |
| 2 | 15/63 = ~0.238 |
| 3 | 20/63 = ~0.317 |
| 4 | 15/63 = ~0.238 |
| 5 | 6/63 = ~0.095 |
| 6 | 1/63 = ~0.016 |

^*^ Microbiome occurrence represents the number of phyllosphere microbiomes in which an ASV is present within each site out of a maximum of six.

^+^ Probabilities are calculated by dividing the number of unique microbiome combinations an ASV could occupy per microbiome occurrence, by the total number of possible combinations.

The number of unique combinations is:

$C(n,r)=\frac{n!}{\left( n-r \right)!r!}$

Probabilities were zero truncated due to the inability to measure undetected microbial taxa.

**Supplementary Table 3:** The observed number of ASVs found at each microbiome occurrence per site

|  | Microbiome occurrence | | | | | |  |
| --- | --- | --- | --- | --- | --- | --- | --- |
| Sites | 1 | 2 | 3 | 4 | 5 | 6 | Total |
| 1 | 1002 | 229 | 150 | 139 | 56 | 211 | 1787 |
| 2 | 971 | 187 | 149 | 90 | 108 | 236 | 1741 |
| 4 | 952 | 222 | 147 | 106 | 119 | 267 | 1813 |
| 5 | 1181 | 209 | 108 | 91 | 95 | 274 | 1958 |
| 6 | 634 | 196 | 142 | 110 | 129 | 143 | 1354 |

**Supplementary Table 4:** Chi-square goodness of fit test results of the occurrence probabilities (Supplementary Table 2) vs the observed number of ASVs (Supplementary Table 3).

|  | X2 | Df | P value |
| --- | --- | --- | --- |
| Site 1 | 5908.6 | 5 | < 2.2x10-6 |
| Site 2 | 6175.1 | 5 | < 2.2x10-6 |
| Site 4 | 6172.8 | 5 | < 2.2x10-6 |
| Site 5 | 8115.8 | 5 | < 2.2x10-6 |
| Site 6 | 3047.2 | 5 | < 2.2x10-6 |

**Supplementary Table 5:** ASV occurrence probabilities predicted for each microbiome occurrence per host group/site

| Microbiome occurrence^*^ | Probability a taxon is found at an occurrence^+^ with zero truncation (mānuka) | Probability a taxon is found at an occurrence^+^ with zero truncation  (non-mānuka) |
| --- | --- | --- |
| 0 | NA | NA |
| 1 | 3/7 = ~0.429 | 3/7 = ~0.429 |
| 2 | 3/7 = ~0.429 | 3/7 = ~0.429 |
| 3 | 1/7 = ~0.143 | 1/7 = ~0.143 |

^*^ Microbiome occurrence represents the number of phyllosphere microbiomes in which an ASV is present, out of a maximum of three per each host group (i.e. mānuka or non-mānuka).

^+^ Probabilities are calculated by dividing the number of unique microbiome combinations an ASV could occupy per microbiome occurrence, by the total number of possible combinations.

The number of unique combinations is:

$C(n,r)=\frac{n!}{\left( n-r \right)!r!}$

Probabilities were zero truncated due to the inability to measure undetected microbial taxa.

**Supplementary Table 6:** The observed number of ASVs found at each microbiome occurrence within mānuka and non-mānuka microbiomes per site

|  | Microbiome occurrence | | | | | | | |
| --- | --- | --- | --- | --- | --- | --- | --- | --- |
|  | Mānuka | | | | Non-mānuka | | | |
|  | 1 | 2 | 3 | Total | 1 | 2 | 3 | Total |
| Site 1 | 461 | 173 | 449 | 1083 | 865 | 162 | 223 | 1250 |
| Site 2 | 460 | 166 | 446 | 1072 | 815 | 226 | 237 | 1278 |
| Site 4 | 268 | 174 | 476 | 918 | 959 | 288 | 293 | 1540 |
| Site 5 | 374 | 130 | 457 | 961 | 1111 | 189 | 304 | 1604 |
| Site 6 | 312 | 153 | 464 | 929 | 564 | 166 | 163 | 893 |

**Supplementary Table 7:** Chi-square goodness of fit test results of the occurrence probabilities (Supplementary Table 5) vs the observed number of ASVs (Supplementary Table 6).

|  | Mānuka | | | | Non-mānuka | | | |
| --- | --- | --- | --- | --- | --- | --- | --- | --- |
|  | X^2^ | Df | P value | JSD^+^ | X^2^ | Df | P value | JSD^+^ |
| Site 1 | 742.41 | 2 | < 0.001 | 0.0959 | 474.16 | 2 | < 0.001 | 0.0849 |
| Site 2 | 747.44 | 2 | < 0.001 | 0.0985 | 335.63 | 2 | < 0.001 | 0.0558 |
| Site 4 | 1069.2 | 2 | < 0.001 | 0.1254 | 369.35 | 2 | < 0.001 | 0.0506 |
| Site 5 | 940.93 | 2 | < 0.001 | 0.1266 | 646.84 | 2 | < 0.001 | 0.0927 |
| Site 6 | 996.54 | 2 | < 0.001 | 0.1235 | 218.43 | 2 | < 0.001 | 0.0514 |

**Supplementary Table 8:** Minimum, maximum, and average relative abundance (%) of phyla in all phyllosphere microbiome samples.

| Phylum | minimum | maximum | Average |
| --- | --- | --- | --- |
| Abditibacteriota | 0 | 2.5014 | 0.1839 |
| Acidobacteriota | 10.1446 | 34.8733 | 23.7166 |
| Actinobacteriota | 0 | 2.1631 | 0.3749 |
| Armatimonadota | 0 | 2.7995 | 0.7518 |
| Bacteroidota | 0.11 | 15.7523 | 4.385 |
| Bdellovibrionota | 0 | 2.7665 | 0.8514 |
| Chloroflexi | 0 | 0.0614 | 0.0018 |
| Cyanobacteria | 0 | 2.1603 | 0.1869 |
| Deinococcota | 0 | 0.2735 | 0.0342 |
| Desulfobacterota | 0 | 0.0338 | 0.001 |
| Firmicutes | 0 | 1.136 | 0.0648 |
| Myxococcota | 0 | 1.3368 | 0.2839 |
| Nitrospirota | 0 | 0.0272 | 0.0008 |
| Planctomycetota | 0.4568 | 6.7188 | 2.2585 |
| Proteobacteria | 35.9113 | 80.0674 | 61.4325 |
| Verrucomicrobiota | 1.198 | 11.6899 | 5.472 |

**Supplementary Table 9:** Minimum, maximum, and average relative abundance (%) of phyla in the phyllosphere microbiome of mānuka, kānuka, tawiniwini, and toatoa.

| Phylum | Host_species | minimum | maximum | Average |
| --- | --- | --- | --- | --- |
| Abditibacteriota | Kānuka | 0 | 0.1915 | 0.0341 |
|  | Mānuka | 0 | 0.2579 | 0.0243 |
|  | Tawiniwini | 0.1749 | 2.2209 | 0.7115 |
|  | Toatoa | 0 | 0.5474 | 0.111 |
| Acidobacteriota | Kānuka | 9.7824 | 20.6749 | 14.814 |
|  | Mānuka | 19.2458 | 28.8484 | 22.934 |
|  | Tawiniwini | 9.1709 | 21.9948 | 17.9094 |
|  | Toatoa | 11.3846 | 26.7284 | 21.5378 |
| Actinobacteriota | Kānuka | 0.0196 | 0.8684 | 0.4348 |
|  | Mānuka | 0 | 0.1948 | 0.0435 |
|  | Tawiniwini | 0.2199 | 1.1704 | 0.601 |
|  | Toatoa | 0.1366 | 1.6712 | 0.6697 |
| Armatimonadota | Kānuka | 0.0958 | 0.4972 | 0.329 |
|  | Mānuka | 0 | 0.7552 | 0.2778 |
|  | Tawiniwini | 0.4732 | 1.752 | 1.1389 |
|  | Toatoa | 0.1489 | 2.1937 | 1.4581 |
| Bacteroidota | Kānuka | 3.3189 | 7.6052 | 5.1251 |
|  | Mānuka | 0.1027 | 4.9919 | 1.7096 |
|  | Tawiniwini | 4.7012 | 12.5753 | 8.6379 |
|  | Toatoa | 0.2727 | 5.7385 | 3.1931 |
| Bdellovibrionota | Kānuka | 0.2936 | 2.5594 | 0.8666 |
|  | Mānuka | 0 | 1.2142 | 0.6962 |
|  | Tawiniwini | 0.3162 | 1.3083 | 0.7612 |
|  | Toatoa | 0.1092 | 1.343 | 0.7363 |
| Chloroflexi | Kānuka | 0 | 0 | 0 |
|  | Mānuka | 0 | 0 | 0 |
|  | Tawiniwini | 0 | 0.0545 | 0.0091 |
|  | Toatoa | 0 | 0 | 0 |
| Cyanobacteria | Kānuka | 0 | 0 | 0 |
|  | Mānuka | 0 | 0 | 0 |
|  | Tawiniwini | 0.0187 | 1.1419 | 0.3915 |
|  | Toatoa | 0.0207 | 1.34 | 0.3931 |
| Deinococcota | Kānuka | 0 | 0.1657 | 0.0391 |
|  | Mānuka | 0 | 0.2466 | 0.0354 |
|  | Tawiniwini | 0 | 0.2399 | 0.04 |
|  | Toatoa | 0 | 0 | 0 |
| Desulfobacterota | Kānuka | 0 | 0.0313 | 0.0052 |
|  | Mānuka | 0 | 0 | 0 |
|  | Tawiniwini | 0 | 0 | 0 |
|  | Toatoa | 0 | 0 | 0 |
| Firmicutes | Kānuka | 0 | 0.0637 | 0.0106 |
|  | Mānuka | 0 | 0.0831 | 0.0049 |
|  | Tawiniwini | 0 | 0.7818 | 0.1747 |
|  | Toatoa | 0 | 0.3163 | 0.0818 |
| Myxococcota | Kānuka | 0 | 1.2062 | 0.4629 |
|  | Mānuka | 0 | 0.5156 | 0.1918 |
|  | Tawiniwini | 0 | 0.3962 | 0.1875 |
|  | Toatoa | 0 | 0.5169 | 0.243 |
| Nitrospirota | Kānuka | 0 | 0.0245 | 0.0041 |
|  | Mānuka | 0 | 0 | 0 |
|  | Tawiniwini | 0 | 0 | 0 |
|  | Toatoa | 0 | 0 | 0 |
| Planctomycetota | Kānuka | 0.6399 | 3.3701 | 1.6742 |
|  | Mānuka | 0.4182 | 4.1757 | 2.0288 |
|  | Tawiniwini | 0.7767 | 1.5779 | 1.2041 |
|  | Toatoa | 0.7444 | 5.265 | 2.5685 |
| Proteobacteria | Kānuka | 63.5682 | 82.0143 | 73.5 |
|  | Mānuka | 63.6813 | 72.4008 | 67.8198 |
|  | Tawiniwini | 48.9659 | 78.531 | 62.6956 |
|  | Toatoa | 53.8798 | 82.6104 | 61.9965 |
| Verrucomicrobiota | Kānuka | 1.1083 | 4.6758 | 2.7003 |
|  | Mānuka | 1.3102 | 8.237 | 4.234 |
|  | Tawiniwini | 2.8855 | 8.9382 | 5.5376 |
|  | Toatoa | 1.7866 | 10.851 | 7.011 |

**Supplementary Table 10:** Kruskal-wallis rank sum test on phylum relative abundance across host species. ‘P adjusted’ represents Benjamini-Hochberg corrected p values. Significant values are in bold.

| Phylum | Statistic | P value | P adjusted |
| --- | --- | --- | --- |
| Abditibacteriota | 18.5538 | 0.0003 | **0.0018** |
| Acidobacteriota | 15.9628 | 0.0012 | **0.0037** |
| Actinobacteriota | 16.1661 | 0.001 | **0.0037** |
| Armatimonadota | 10.205 | 0.0169 | **0.03** |
| Bacteroidota | 20.7632 | 0.0001 | **0.0009** |
| Bdellovibrionota | 0.7171 | 0.8692 | 0.8819 |
| Chloroflexi | 4.8333 | 0.1844 | 0.2459 |
| Cyanobacteria | 28.869 | 0 | **0** |
| Deinococcota | 2.0669 | 0.5586 | 0.6384 |
| Desulfobacterota | 4.8333 | 0.1844 | 0.2459 |
| Firmicutes | 15.1192 | 0.0017 | **0.0046** |
| Myxococcota | 0.6627 | 0.8819 | 0.8819 |
| Nitrospirota | 4.8333 | 0.1844 | 0.2459 |
| Planctomycetota | 4.3673 | 0.2244 | 0.2762 |
| Proteobacteria | 11.8036 | 0.0081 | **0.0162** |
| Verrucomicrobiota | 11.9406 | 0.0076 | **0.0162** |

**Supplementary Table 11:** Summary of alpha diversity in the phyllosphere microbiome of different host species. Values represent the mean and standard deviation of 100 subsamples to the minimum sequencing depth (6192 reads).

| Sample_ID | Richness | | Shannon | | Host species |
| --- | --- | --- | --- | --- | --- |
|  | mean | sd | mean | sd |  |
| Rp_01.1 | 625.53 | 5.9501 | 6.0809 | 0.0114 | Mānuka |
| Rp_01.2 | 723.38 | 7.2122 | 6.2471 | 0.0107 | Mānuka |
| Rp_01.3 | 624.42 | 4.9425 | 6.1138 | 0.0087 | Mānuka |
| Rp_01.4 | 714.93 | 5.5108 | 6.1945 | 0.0104 | Toatoa |
| Rp_01.5 | 471.46 | 5.2309 | 5.646 | 0.0111 | Kānuka |
| Rp_01.6 | 536.25 | 4.4481 | 5.8803 | 0.0104 | Tawiniwini |
| Rp_02.1 | 628 | 5.3314 | 6.1232 | 0.0089 | Mānuka |
| Rp_02.2 | 688.31 | 6.8721 | 6.2201 | 0.01 | Mānuka |
| Rp_02.3 | 614.6 | 5.8205 | 6.1077 | 0.0103 | Mānuka |
| Rp_02.4 | 633.02 | 5.1345 | 6.071 | 0.0099 | Toatoa |
| Rp_02.5 | 573.12 | 4.9304 | 5.954 | 0.0123 | Kānuka |
| Rp_02.6 | 638.61 | 5.253 | 6.1015 | 0.0098 | Tawiniwini |
| Rp_03.2 | 713.31 | 5.6295 | 6.2576 | 0.0088 | Mānuka |
| Rp_03.3 | 674.83 | 6.5691 | 6.1865 | 0.0112 | Mānuka |
| Rp_03.4 | 526.93 | 4.5644 | 5.8565 | 0.0107 | Toatoa |
| Rp_03.5 | 482.7 | 5.1669 | 5.7634 | 0.0109 | Kānuka |
| Rp_03.6 | 697.1 | 6.7293 | 6.1585 | 0.0103 | Tawiniwini |
| Rp_04.1 | 645.77 | 6.6239 | 6.0616 | 0.0117 | Mānuka |
| Rp_04.2 | 616.8 | 5.2724 | 6.0982 | 0.0093 | Mānuka |
| Rp_04.3 | 618.34 | 5.5126 | 6.0832 | 0.0108 | Mānuka |
| Rp_04.4 | 1001.44 | 7.1807 | 6.6253 | 0.0094 | Toatoa |
| Rp_04.5 | 477.01 | 3.2737 | 5.8682 | 0.0086 | Kānuka |
| Rp_04.6 | 721.31 | 6.743 | 6.2067 | 0.0121 | Tawiniwini |
| Rp_05.1 | 684.2 | 6.5056 | 6.2109 | 0.0102 | Mānuka |
| Rp_05.2 | 633.76 | 4.928 | 6.1355 | 0.0094 | Mānuka |
| Rp_05.3 | 528.01 | 4.2247 | 5.9088 | 0.0111 | Mānuka |
| Rp_05.4 | 741.22 | 6.3319 | 6.3221 | 0.0111 | Toatoa |
| Rp_05.5 | 538.29 | 6.1698 | 5.8956 | 0.012 | Kānuka |
| Rp_05.6 | 862.04 | 9.243 | 6.3622 | 0.011 | Tawiniwini |
| Rp_06.1 | 586.26 | 5.0063 | 6.0068 | 0.0101 | Mānuka |
| Rp_06.2 | 635.38 | 6.2098 | 6.1065 | 0.0088 | Mānuka |
| Rp_06.3 | 590.69 | 6.7265 | 5.9222 | 0.0137 | Mānuka |
| Rp_06.4 | 309.33 | 2.4579 | 5.2887 | 0.01 | Toatoa |
| Rp_06.5 | 547.07 | 5.8556 | 5.9409 | 0.0104 | Kānuka |
| Rp_06.6 | 456.59 | 3.062 | 5.6941 | 0.0125 | Tawiniwini |

**Supplementary Table 12:** ANOVA on phyllosphere microbiome alpha diversity across host species.

| Alpha diversity index |  | Df | Sum Sq | Mean Sq | F value | P value |
| --- | --- | --- | --- | --- | --- | --- |
| Shannon | Host species | 3 | 0.3164 | 0.1055 | 2.092 | 0.122 |
|  | Residuals | 31 | 1.5633 | 0.05043 |  |  |
| Richness | Host species | 3 | 84392 | 28131 | 2.089 | 0.122 |
|  | Residuals | 31 | 417358 | 13463 |  |  |

**Supplementary Table 13:** Kruskal-wallis rank sum test on indicator species relative abundance at the phylum level. ‘P adjusted’ represents Benjamini-Hochberg corrected p values. Significant values are in bold.

| Phylum | P adjusted |
| --- | --- |
| Acidobacteriota | **0.0002** |
| Bacteroidota | **0.0004** |
| Proteobacteria | **0.0004** |
| Verrucomicrobiota | **0.0005** |

**Supplementary Table 14:** Kruskal-wallis rank sum test on indicator species relative abundance at the genus level. ‘P adjusted’ represents Benjamini-Hochberg corrected p values. Significant values are in bold.

| Genus | P adjusted |
| --- | --- |
| 1174-901-12 | **0.0002** |
| Acidiphilium | **0.0011** |
| Aurantisolimonas | **0.0005** |
| Bryocella | **0.0001** |
| Edaphobacter | **0.0253** |
| Granulicella | **0.0003** |
| LD29 | **0.0009** |
| Methylocella | **0.0002** |
| PMMR1 | **0.0277** |
| Sphingomonas | **0.0253** |
| Terriglobus | **0.007** |
| Unknown | **0.0009** |

**Supplementary Table 15:** Pairwise comparisons of betadisper test. Significant values are in bold.

| Host species comparison | Bray-Curtis (p value) | Jaccard (p value) |
| --- | --- | --- |
| Mānuka-Kānuka | 0.399 | **0.001** |
| Mānuka-Tawiniwini | **0.001** | **0.001** |
| Mānuka-Toatoa | **0.001** | **0.001** |
| Kānuka-Tawiniwini | **0.008** | 0.189 |
| Kānuka-Toatoa | **0.001** | **0.02** |
| Tawiniwini-Toatoa | 0.077 | 0.109 |

**Supplementary Table 16:** Normalised stochasticity ratios (NST) of phyllosphere community assembly based on taxonomy.

| Host species | NST |
| --- | --- |
| Mānuka | 0.13 |
| Kānuka | 0.13 |
| Tawiniwini | 0.34 |
| Toatoa | 0.48 |

**Supplementary Table 17:** Permanova on normalised stochasticity ratios (NST) of phyllosphere community assembly based on taxonomy.

| Host species comparison | P value |
| --- | --- |
| Mānuka-Kānuka | 0.860 |
| Mānuka-Tawiniwini | **0.001** |
| Mānuka-Toatoa | **0.001** |
| Kānuka-Tawiniwini | **0.001** |
| Kānuka-Toatoa | **0.005** |
| Tawiniwini-Toatoa | 0.074 |

**Supplementary Table 18:** Mantel analyses for different partitions of the phyllosphere microbiome of all host species. Mantel tests performed on community dissimilarity (Bray-Curtis, relative abundance; Jaccard, presence/absence) and Euclidean distance. Significant values are in bold.

| Community partition | All phyllosphere samples | |
| --- | --- | --- |
|  | Mantel r | P value |
| High abundance  (>0.001) | 0.079 | 0.192 |
| Entire community | 0.05 | 0.296 |
| Low abundance  (<0.001) | 0.1527 | 0.059 |
| Presence/absence | 0.09 | 0.179 |

**Supplementary Table 19:** Mantel analyses for different partitions of the mānuka and non-mānuka phyllosphere microbiome. Mantel tests performed on community dissimilarity (Bray-Curtis, relative abundance; Jaccard, presence/absence) and Euclidean distance. Significant values are in bold.

| Community partition | Mānuka | | Kānuka | | Toatoa | | Tawiniwini | |
| --- | --- | --- | --- | --- | --- | --- | --- | --- |
|  | Mantel r | P value | Mantel r | P value | Mantel r | P value | Mantel r | P value |
| High abundance (>0.001) | **0.388** | **0.001** | 0.4714 | 0.1514 | **0.7964** | **0.02361** | **0.6786** | **0.0097** |
| Entire community | **0.3174** | **0.009** | 0.5393 | 0.1181 | **0.7786** | **0.0333** | 0.4679 | 0.0750 |
| Low abundance (<0.001) | **0.4457** | **0.001** | 0.725 | 0.0597 | 0.6714 | 0.0680 | 0.3536 | 0.1167 |
| Presence/absence | **0.4092** | **0.001** | 0.3821 | 0.1763 | 0.6429 | 0.0930 | 0.4214 | 0.1014 |

**Supplementary Table 20:** Minimum, maximum, and average relative abundance (%) of phyla in the surface soil microbiome.

| Phylum | Minimum | Maximum | Average |
| --- | --- | --- | --- |
| Acidobacteriota | 8.8107 | 24.3939 | 15.1887 |
| Actinobacteriota | 0.0335 | 9.443 | 2.3567 |
| Armatimonadota | 0 | 0.844 | 0.2658 |
| Bacteroidota | 9.9583 | 38.4848 | 17.9208 |
| Bdellovibrionota | 0.0864 | 1.827 | 0.7169 |
| Chloroflexi | 0.4414 | 11.2985 | 2.4383 |
| Crenarchaeota | 0 | 0.1949 | **0.0213** |
| Cyanobacteria | 0 | 1.1586 | 0.3799 |
| Deinococcota | 0 | 0.1079 | **0.0045** |
| Dependentiae | 0.0721 | 4.5571 | 0.756 |
| Desulfobacterota | 0 | 0.0656 | **0.0027** |
| Elusimicrobiota | 0 | 0.4206 | **0.0175** |
| Entotheonellaeota | 0 | 0.0797 | **0.0056** |
| Fibrobacterota | 0 | 0.1402 | **0.0072** |
| Firmicutes | 0 | 0.9025 | 0.1441 |
| Gemmatimonadota | 0 | 0.3155 | 0.0838 |
| Methylomirabilota | 0 | 0.1129 | **0.0078** |
| Micrarchaeota | 0 | 0.011 | **0.0005** |
| Myxococcota | 0.1844 | 3.9418 | 1.5172 |
| Nanoarchaeota | 0 | 0.0117 | **0.0005** |
| Nitrospirota | 0 | 0.2435 | **0.0381** |
| Planctomycetota | 0.3352 | 11.819 | 6.0289 |
| Proteobacteria | 26.3768 | 44.612 | 34.2122 |
| Spirochaetota | 0 | 0.3991 | 0.0789 |
| Verrucomicrobiota | 10.8616 | 24.6129 | 17.806 |

**Supplementary Table 21:** Mantel analyses for different partitions of surface soil microbial communities. Mantel tests performed on community dissimilarity (Bray-Curtis, relative abundance; Jaccard, presence/absence) and Euclidean distance.

| Community partition | All samples | |
| --- | --- | --- |
|  | Mantel r | P value |
| High abundance (>0.001) | -0.0420 | 0.572 |
| Entire community | -0.0377 | 0.600 |
| Low abundance (<0.001) | 0.0019 | 0.471 |
| Presence/absence | 0.0047 | 0.465 |

**Supplementary Table 22:** Summary of alpha diversity across phyllosphere and surface soil samples. Values represent the mean and standard deviation of 100 subsamples to the minimum sequencing depth (5966 reads).

| SampleID | Richness | | Shannon | | Type |  |
| --- | --- | --- | --- | --- | --- | --- |
|  | mean | sd | mean | sd |  | Host species |
| Rp_01.1 | 623.85 | 4.8812 | 6.0805 | 0.0108 | Leaf | Mānuka |
| Rp_01.1S | 588.29 | 5.6377 | 6.0312 | 0.011 | Soil | Mānuka |
| Rp_01.2 | 720.36 | 6.0244 | 6.2436 | 0.01 | Leaf | Mānuka |
| Rp_01.3 | 622.48 | 5.3 | 6.1152 | 0.0102 | Leaf | Mānuka |
| Rp_01.4 | 710.26 | 6.7981 | 6.1904 | 0.0107 | Leaf | Toatoa |
| Rp_01.4S | 370.63 | 3.6671 | 5.6009 | 0.0097 | Soil | Toatoa |
| Rp_01.5 | 469.8 | 4.4969 | 5.6452 | 0.0115 | Leaf | Kānuka |
| Rp_01.5S | 524.18 | 4.7893 | 5.9033 | 0.009 | Soil | Kānuka |
| Rp_01.6 | 533.7 | 4.8814 | 5.8754 | 0.0107 | Leaf | Tawiniwini |
| Rp_01.6S | 650.47 | 6.4939 | 6.1265 | 0.0097 | Soil | Tawiniwini |
| Rp_02.1 | 628.01 | 5.9365 | 6.1245 | 0.0096 | Leaf | Mānuka |
| Rp_02.1S | 495.11 | 4.5945 | 5.8648 | 0.0077 | Soil | Mānuka |
| Rp_02.2 | 684.86 | 6.6606 | 6.2169 | 0.0098 | Leaf | Mānuka |
| Rp_02.3 | 612.56 | 6.4375 | 6.1054 | 0.0102 | Leaf | Mānuka |
| Rp_02.4 | 629.98 | 5.545 | 6.0713 | 0.0101 | Leaf | Toatoa |
| Rp_02.4S | 566.55 | 4.6174 | 6.038 | 0.01 | Soil | Toatoa |
| Rp_02.5 | 570.64 | 5.0503 | 5.9492 | 0.0111 | Leaf | Kānuka |
| Rp_02.5S | 677.12 | 6.5185 | 6.1814 | 0.0088 | Soil | Kānuka |
| Rp_02.6 | 636.28 | 5.5761 | 6.1013 | 0.0107 | Leaf | Tawiniwini |
| Rp_02.6S | 624.61 | 6.3864 | 6.0983 | 0.0104 | Soil | Tawiniwini |
| Rp_03.1S | 552.19 | 5.3252 | 5.92 | 0.0104 | Soil | Mānuka |
| Rp_03.2 | 710.22 | 6.6265 | 6.2544 | 0.0108 | Leaf | Mānuka |
| Rp_03.3 | 672.08 | 6.6434 | 6.1826 | 0.0102 | Leaf | Mānuka |
| Rp_03.4 | 523.74 | 4.0741 | 5.8558 | 0.0109 | Leaf | Toatoa |
| Rp_03.4S | 496.4 | 4.1317 | 5.8506 | 0.0094 | Soil | Toatoa |
| Rp_03.5 | 480.39 | 4.8636 | 5.7616 | 0.0097 | Leaf | Kānuka |
| Rp_03.5S | 382.44 | 4.1593 | 5.5905 | 0.0088 | Soil | Kānuka |
| Rp_03.6 | 691.88 | 8.3995 | 6.1568 | 0.0118 | Leaf | Tawiniwini |
| Rp_03.6S | 196.33 | 1.1015 | 4.9855 | 0.0086 | Soil | Tawiniwini |
| Rp_04.1 | 644.47 | 6.2884 | 6.0621 | 0.0115 | Leaf | Mānuka |
| Rp_04.1S | 537.97 | 5.016 | 5.9771 | 0.0087 | Soil | Mānuka |
| Rp_04.2 | 614.96 | 5.3709 | 6.0971 | 0.0103 | Leaf | Mānuka |
| Rp_04.3 | 616.62 | 6.1262 | 6.08 | 0.0103 | Leaf | Mānuka |
| Rp_04.4 | 996.91 | 7.3417 | 6.623 | 0.0086 | Leaf | Toatoa |
| Rp_04.4S | 545.06 | 5.1006 | 5.942 | 0.01 | Soil | Toatoa |
| Rp_04.5 | 476.4 | 4.3647 | 5.867 | 0.0081 | Leaf | Kānuka |
| Rp_04.5S | 697.11 | 5.944 | 6.2477 | 0.0097 | Soil | Kānuka |
| Rp_04.6 | 718.02 | 7.0324 | 6.2045 | 0.0098 | Leaf | Tawiniwini |
| Rp_04.6S | 796.67 | 7.4523 | 6.3098 | 0.0113 | Soil | Tawiniwini |
| Rp_05.1 | 681.02 | 6.818 | 6.206 | 0.0109 | Leaf | Mānuka |
| Rp_05.1S | 497.1 | 4.7001 | 5.8491 | 0.0102 | Soil | Mānuka |
| Rp_05.2 | 631.03 | 5.5221 | 6.1334 | 0.01 | Leaf | Mānuka |
| Rp_05.3 | 526.65 | 4.3073 | 5.9091 | 0.0097 | Leaf | Mānuka |
| Rp_05.4 | 737.33 | 5.4663 | 6.319 | 0.0086 | Leaf | Toatoa |
| Rp_05.4S | 622.79 | 5.7547 | 6.1071 | 0.0102 | Soil | Toatoa |
| Rp_05.5 | 537.35 | 6.1814 | 5.8934 | 0.011 | Leaf | Kānuka |
| Rp_05.5S | 549.28 | 5.6641 | 5.9723 | 0.0103 | Soil | Kānuka |
| Rp_05.6 | 859.47 | 10.4374 | 6.3616 | 0.0124 | Leaf | Tawiniwini |
| Rp_05.6S | 579.92 | 5.8148 | 6.0754 | 0.0095 | Soil | Tawiniwini |
| Rp_06.1 | 584.28 | 5.1111 | 6.0046 | 0.0098 | Leaf | Mānuka |
| Rp_06.1S | 526.12 | 4.961 | 5.9332 | 0.0108 | Soil | Mānuka |
| Rp_06.2 | 633.92 | 6.4771 | 6.1048 | 0.0094 | Leaf | Mānuka |
| Rp_06.3 | 587.7 | 5.6667 | 5.9203 | 0.0109 | Leaf | Mānuka |
| Rp_06.4 | 308.77 | 2.5736 | 5.2885 | 0.01 | Leaf | Toatoa |
| Rp_06.4S | 637.33 | 6.189 | 6.1358 | 0.0097 | Soil | Toatoa |
| Rp_06.5 | 545.12 | 4.9712 | 5.9401 | 0.0106 | Leaf | Kānuka |
| Rp_06.5S | 595.87 | 5.5005 | 6.073 | 0.0096 | Soil | Kānuka |
| Rp_06.6 | 455.55 | 3.3886 | 5.6944 | 0.0107 | Leaf | Tawiniwini |
| Rp_06.6S | 513.18 | 4.6196 | 5.9125 | 0.01 | Soil | Tawiniwini |

**References**

Allen, R.*, et al.* 1992, 'Ecology of Kunzea ericoides (A. Rich.) J. Thompson (Kānuka) in east Otago, New Zealand', *New Zealand journal of botany*, vol. 30, no. 2, pp. 135-149.

Burrows, C. 1973, 'The ecological niches of Leptospermum scoparium and L. ericoides (Angiospermae: Myrtaceae)', vol. no. pp.

Bush, C. M.*, et al.* 2009, 'The phylogeny, biogeography and morphological evolution of Gaultheria (Ericaceae) from Australia and New Zealand', *Australian Systematic Botany*, vol. 22, no. 4, pp. 229-242.

de Lange, P. J. 2014, 'A revision of the New Zealand Kunzea ericoides (Myrtaceae) complex', *PhytoKeys*, vol. no. 40, pp. 1.

Johnson, C. T. 1980, 'leaf anatomy of Leptospermum Forst. (Myrtaceae)', *Australian journal of botany*, vol. 28, no. 1, pp. 77-104.

Marden, M.*, et al.* 2021, 'Species-specific basic stem-wood densities for twelve indigenous forest and shrubland species of known age, New Zealand', *New Zealand Journal of Forestry Science*, vol. 51, no. pp.

Stephens, J.*, et al.* 2005, *A review of Leptospermum scoparium (Myrtaceae) in New Zealand*,

Wardle, P. 1969, 'Biological flora of New Zealand', *New Zealand Journal of Botany*, vol. 7, no. 1, pp. 76-95.

Whitehead, D.*, et al.* 2004, 'Characteristics of photosynthesis and stomatal conductance in the shrubland species mānuka (Leptospermum scoparium) and kānuka (Kunzea ericoides) for the estimation of annual canopy carbon uptake', *Tree Physiology*, vol. 24, no. 7, pp. 795-804.

1. Stephens, J.*, et al.* 2005, *A review of Leptospermum scoparium (Myrtaceae) in New Zealand*, [↑](#footnote-ref-1)
2. de Lange, P. J. 2014, 'A revision of the New Zealand Kunzea ericoides (Myrtaceae) complex', *PhytoKeys*, vol. no. 40, pp. 1. [↑](#footnote-ref-2)
3. Bush, C. M.*, et al.* 2009, 'The phylogeny, biogeography and morphological evolution of Gaultheria (Ericaceae) from Australia and New Zealand', *Australian Systematic Botany*, vol. 22, no. 4, pp. 229-242. [↑](#footnote-ref-3)
4. Wardle, P. 1969, 'Biological flora of New Zealand', *New Zealand Journal of Botany*, vol. 7, no. 1, pp. 76-95. [↑](#footnote-ref-4)
5. Burrows, C. 1973, 'The ecological niches of Leptospermum scoparium and L. ericoides (Angiospermae: Myrtaceae)', vol. no. pp. [↑](#footnote-ref-5)
6. Allen, R.*, et al.* 1992, 'Ecology of Kunzea ericoides (A. Rich.) J. Thompson (Kānuka) in east Otago, New Zealand', *New Zealand journal of botany*, vol. 30, no. 2, pp. 135-149. [↑](#footnote-ref-6)
7. Whitehead, D.*, et al.* 2004, 'Characteristics of photosynthesis and stomatal conductance in the shrubland species mānuka (Leptospermum scoparium) and kānuka (Kunzea ericoides) for the estimation of annual canopy carbon uptake', *Tree Physiology*, vol. 24, no. 7, pp. 795-804. [↑](#footnote-ref-7)
8. Johnson, C. T. 1980, 'leaf anatomy of Leptospermum Forst. (Myrtaceae)', *Australian journal of botany*, vol. 28, no. 1, pp. 77-104. [↑](#footnote-ref-8)
9. Marden, M.*, et al.* 2021, 'Species-specific basic stem-wood densities for twelve indigenous forest and shrubland species of known age, New Zealand', *New Zealand Journal of Forestry Science*, vol. 51, no. pp. [↑](#footnote-ref-9)
10. Marden, M.*, et al.* 2021, 'Species-specific basic stem-wood densities for twelve indigenous forest and shrubland species of known age, New Zealand', *New Zealand Journal of Forestry Science*, vol. 51, no. pp. [↑](#footnote-ref-10)
